# Supplementary material for: Synthesis and Antimicrobial Activity of Phosphonopeptide Derivatives Incorporating Single and Dual Inhibitors
Source: Molecules. 2020 Mar 28;25(7):1557. doi: 10.3390/molecules25071557 (PMC7180716; doi:10.3390/molecules25071557)
Supplement: Supplementary file 1 [file molecules-25-01557-s001.pdf]

*Supplementary Information*

# Synthesis and Antimicrobial Activity of Phosphonopeptide Derivatives Incorporating Single and Dual Inhibitors

## 1. Full Synthesis and Compound Characterization

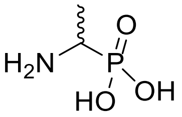

*1-Aminoethylphosphonic Acid or D/L-fosfalin (2-DL)*

To suspension of *N*-phenylthiourea (100.0 mmol, 15.2 g) in glacial acetic acid (50 mL), acetaldehyde (130.0 mmol, 7.40 mL) was added dropwise, followed by triphenyl phosphite (100.0 mmol, 27.0 mL). The mixture was stirred at room temperature for 5 mins, then refluxed at 80 °C for 1 hr until a clear solution was obtained. A mixture of glacial acetic acid (5 mL) and hydrochloric acid (37%, 50 mL) was added and the reaction was refluxed overnight. The solution was cooled to room temperature and concentrated in vacuo to afford a brown slurry. Absolute ethanol (150 mL) was added while stirring and the resulting off-white solid was collected by filtration and dried in a desiccator containing phosphorus(V) oxide. The crude solid was recrystallized from hot water/ethanol to afford **2-DL** as white crystals, as a mixture of enantiomers (12.2 g, 98 mmol, 98%); m.p. 271–274 °C (sublim); $\bar{\upsilon}_{max}$/cm$^{-1}$ 2910 (br OH), 1532 (NH bend), 1143 (P = O), 1035 (P-O-C), 930 (P-OH); $^{1}$H NMR (300 MHz, $D_2O$) $\delta_H$ 1.40 (3H, dd, $^{3}J_{H\text{-}P}$ = 14.7 Hz, $^{3}J_{H\text{-}H}$ = 7.2 Hz, C**H**$_3$), 3.33 (1H, m, C**H**); $^{13}$C NMR (75 MHz, $D_2O$) $\delta_C$ 13.5 (d, $^{2}J_{C\text{-}P}$ = 2.6 Hz, **C**H$_3$), 44.7 (d, $^{1}J_{C\text{-}P}$ = 144.2 Hz, **C**H); $^{31}$P-$^{1}$H$_{decoup}$ NMR (121 MHz, $D_2O$) $\delta_P$ 14.2; *m/z* (ESI) calcd for $(C_2H_9NO_3P)^+$, MH$^+$: 126.0, found 126.1; CHN (Found: C, 19.45; H, 6.48; N, 11.18. $C_2H_8NO_3P$ requires C, 19.21; H, 6.45; N, 11.20%).

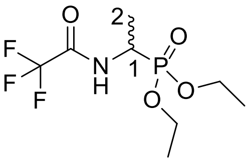

**Diethyl (1-(2,2,2-trifluoroacetamido)ethyl)phosphonate or trifluoroacetyl-D/L-Fos diethyl ester (8)**. 1-Aminoethylphosphonic acid (**2-DL**) (51.7 mmol, 6.5 g) was added to a mixture of trifluoroacetic acid (65.3 mmol, 5 mL) and trifluoroacetic anhydride (177.4 mmol, 25 mL). The solution was stirred and refluxed at 60 °C for 1 hour, then cooled to room temperature and triethyl orthoformate (901.8 mmol, 150 mL) was added dropwise. The solution was refluxed at 110 °C for 2 hours, then cooled to room temperature. The solution was concentrated in vacuo to afford a brown solid, which was re-dissolved in DCM and purified by column chromatography using a gradient elution (DCM (100) to DCM/MeOH (95:5)) to give **8** as an off-white solid, a mixture of enantiomers (11.4 g, 41.0 mmol, 80%); m.p. 101 – 103 °C (sublim) (lit. m.p. 101—102 °C); $\bar{\upsilon}_{max}$/cm$^{-1}$ 3202 (NH), 1715 (C = O), 1565 (NH bend), 1210 (P = O), 1011 (P-O-C), 968 (P-O-C); $^{1}$H NMR (300 MHz, $CDCl_3$) $\delta_H$ 1.24 (3H, t, $^{3}J_{H\text{-}H}$ = 7.2 Hz, OCH$_2$C**H**$_3$), 1.27 (3H, t, $^{3}J_{H\text{-}H}$ = 7.2 Hz, OCH$_2$C**H**$_3$), 1.38 (3H, dd, $^{3}J_{H\text{-}P}$ = 16.5 Hz, $^{3}J_{H\text{-}H}$ = 7.2 Hz, C**H**$_3$-2), 4.06 (4H, m, 2 x OC**H**$_2$CH$_3$), 4.39 (1H, m, C**H**-1), 8.00 (1H, d, $^{3}J_{H\text{-}H}$ = 6.0 Hz, N**H**); $^{13}$C NMR (75 MHz, $CDCl_3$) $\delta_C$ 14.8 (**C**H$_3$-2), 16.2 (d, $^{3}J_{C\text{-}P}$ = 2.3 Hz, OCH$_2$**C**H$_3$), 16.3 (d, $^{3}J_{C\text{-}P}$ = 2.3 Hz, OCH$_2$**C**H$_3$), 41.8 (d, $^{1}J_{C\text{-}P}$ = 159.1 Hz, **C**H-1), 62.8 (d, $^{2}J_{C\text{-}P}$ = 7.0 Hz, O**C**H$_2$CH$_3$), 63.2 (d, $^{2}J_{C\text{-}P}$ = 7.1 Hz, O**C**H$_2$CH$_3$), 115.9 (q, $^{1}J_{C\text{-}F}$ = 285.8 Hz, **C**F$_3$), 156.9 (q, $^{2}J_{C\text{-}F}$ = 5.8 Hz, **C** = O); $^{31}$P-$^{1}$H$_{decoup}$ NMR (121 MHz, $CDCl_3$) $\delta_P$ 23.0; $^{19}$F-$^{1}$H$_{decoup}$ NMR (282 MHz, $CDCl_3$) $\delta_P$ -75.5; *m/z* (ESI) calcd for $(C_8H_{16}F_3NO_4P)^+$, MH$^+$: 278.1, found 278.1.

lit. m.p.; Kudzin, Z.H; Luczak, J. *Synthesis*. **1995**, 509-511 (DOI: 10.1055/s-1995-3952).

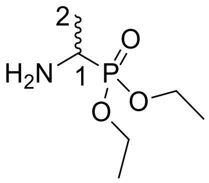

**Diethyl 1-aminoethylphosphonate or D/L-Fos diethyl ester (9)**. Diethyl (1-(2,2,2-trifluoroacetamido)ethyl)phosphonate (**8**) (20.0 mmol, 5.6 g) was dissolved in ethanol (200 ml) and excess sodium borohydride (200.0 mmol, 7.7 g) was added slowly with stirring. The resulting mixture was stirred at room temperature for 1 hour, then heated at reflux for 4 hours. The mixture was cooled to room temperature and the solvent was removed *in vacuo* to afford a white solid, which was dissolved in saturated $NaHCO_3$ (96 g/L) (60 mL) with the addition of 10% aqueous $K_2CO_3$ (20 mL). The product was extracted into DCM (6 x 30 mL) and dried over $MgSO_4$. The filtrate was concentrated *in vacuo* to afford a pale yellow liquid and purified by column chromatography using a gradient elution (DCM (100) to DCM/MeOH (90:10)) to afford **9** as a yellow liquid, a mixture of enantiomers (3.5 g, 19.3 mmol, 97%); $\bar{\upsilon}_{max}$/cm$^{-1}$ 3431 (NH), 1215 (P = O), 1020 (P-O-C), 967 (P-O-C); $^1$H NMR (300 MHz, $CDCl_3$) $\delta_H$ 1.26 (6H, t, $^3J_{H-H}$ = 7.2 Hz, 2 x $OCH_2$**$CH_3$**), 1.34 (3H, dd, $^3J_{H-P}$ = 17.7 Hz, $^3J_{H-H}$ = 7.2 Hz, **$CH_3$**-2), 1.68 (2H, br, N**$H_2$**), 3.02-3.12 (1H, m, **CH**-1), 4.06-4.17 (4H, m, 2 x O**$CH_2$**$CH_3$); $^{13}$C NMR (75 MHz, $CDCl_3$) $\delta_C$ 16.4 ($OCH_2$**$CH_3$**), 16.5 ($OCH_2$**$CH_3$**), 17.2 (**C**$H_3$-2), 44.2 (d, $^1J_{C-P}$ = 148.5 Hz, **C**H-1), 62.1 (d, $^2J_{C-P}$ = 7.5 Hz, O**C**$H_2CH_3$), 62.1 (d, $^2J_{C-P}$ = 7.5 Hz, O**C**$H_2CH_3$); $^{31}$P-$^1$H$_{decoup}$ NMR (121 MHz, $CDCl_3$) $\delta_P$ 29.6; HRMS (NSI) calcd for $(C_6H_{17}NO_3P)^+$, MH$^+$: 204.0760, found 204.0762. LCMS purity >95% (C-18 reversed phase, MeOH-$H_2O$).

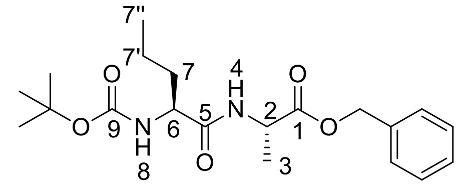

**(*S*)-Benzyl 2-((*S*)-2-((tert-butoxycarbonyl)amino)pentanamido) propanoate or Boc-L-Nva-L-Ala-OBzl (16a)**. General peptide coupling method was followed, using Boc-L-Nva-OH (**15a**) (10.0 mmol, 2.17 g) in dry THF and L-alanine benzyl ester *p*-tosylic acid (10.0 mmol, 3.52 g) in dry DCM. The yellow crude liquid was purified by column chromatography (40-60 petrol/ethyl acetate (7:3)) to give **16a** as an off-white solid (2.40 g, 6.3 mmol, 63%); m.p. 60 – 63 °C; $\bar{\upsilon}_{max}$/cm$^{-1}$ 3332 (NH), 1743 (C = O), 1655 (br C = O), 1527 (NH bend), 1245 (C-O), 1162 (C-O); $^1$H NMR (300 MHz, $CDCl_3$) $\delta_H$ 0.83 (3H, t, $^3J_{H-H}$ = 9.0 Hz, **$CH_3$**-7''), 1.25-1.31 (2H, m, **$CH_2$**-7'), 1.34 (3H, d, $^3J_{H-H}$ = 6.0 Hz, **$CH_3$**-3), 1.36 (9H, s, C(C**$H_3$**)$_3$), 1.42-1.54 (1H, m, **CH$_{a/b}$**-7), 1.64-1.73 (1H, m, **CH$_{a/b}$**-7), 4.02 (1H, m, **CH**-6), 4.54 (1H, pentet, $^3J_{H-H}$ = 6.0 Hz, **CH**-2), 4.96 (1H, d, $^3J_{H-H}$ = 9.0 Hz, N**H**-8), 5.07 (1H, d, $^2J_{H-H}$ = 12.0 Hz, O**CH$_{a/b}$**Ar), 5.12 (1H, d, $^2J_{H-H}$ = 12.0 Hz, O**CH$_{a/b}$**Ar), 6.56 (1H, d, $^3J_{H-H}$ = 6.0 Hz, N**H**-4), 7.27 (5H, m, 5 x **CH$_{Ar}$**); $^{13}$C NMR (75 MHz, $CDCl_3$) $\delta_C$ 12.7 (**C**$H_3$-7''), 17.3 (**C**$H_3$-3), 17.8 (**C**$H_2$-7'), 27.3 (C(**C**$H_3$)$_3$), 33.7 (**C**$H_2$-7), 47.1 (**C**H-2), 53.4 (**C**H-6), 66.1(O**C**$H_2$Ar), 79.0 (**C**($CH_3$)$_3$), 127.1-127.6 (**C**H$_{Ar}$), 134.3 (**C**H$_{Ar}$ quat.), 154.6 (**C** = O-9), 170.8 (**C** = O-5), 171.5 (**C** = O-1); HRMS (NSI) calcd for $(C_{20}H_{31}N_2O_5)^+$, MH$^+$: 379.2227, found 379.2222; CHN (Found: C, 63.75; H, 8.37; N, 7.86. $C_{20}H_{30}N_2O_5$ requires C, 63.47; H, 7.99; N, 7.40%).

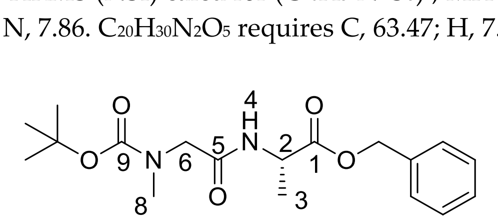

**(*S*)-Benzyl 2-(2-((*tert*-butoxycarbonyl)(methyl)amino)acetamido) propanoate or Boc-Sar-L-Ala-OBzl (16b)**. General peptide coupling method was followed, using Boc-Sar-OH (**15b**) (15.0 mmol, 2.84 g) in dry THF and L-alanine benzyl ester *p*-tosylic acid (15.0 mmol, 5.27 g) in dry DCM. The yellow crude liquid was purified by column chromatography (40-60petrol/ethyl acetate (1:1)) to give **16b** as a colourless liquid (3.93 g, 11 mmol, 75%); $\bar{\upsilon}_{max}$/cm$^{-1}$ 3311 (NH), 1742 (C = O), 1670 (C = O), 1666 (C = O), 1536 (NH bend), 1242 (C-O), 1145 (C-O); $^1$H NMR (300 MHz, $CDCl_3$) $\delta_H$ 1.35 (3H, t, $^3J_{H-H}$ = 6.0

Hz, $CH_3$-3), 1.39 (9H, s, $C(CH_3)_3$), 2.85 (3H, s, $CH_3$-8), 3.72 (1H, d, $^2J_{H-H}$ = 15.0 Hz, $CH_{a/b}$-6), 3.88 (1H, d, $^2J_{H-H}$ = 15.0 Hz, $CH_{a/b}$-6), 4.58 (1H, pentet, $^3J_{H-H}$ = 6.0 Hz, **CH**-2), 5.08 (1H, d, $^2J_{H-H}$ = 12.0 Hz, $OCH_{a/b}Ar$), 5.13 (1H, d, $^2J_{H-H}$ = 12.0 Hz, $OCH_{a/b}Ar$), 6.51 (1H, br, N**H**-4), 7.25-7.29 (5H, m, 5 x $CH_{Ar}$); $^{13}$C NMR (75 MHz, $CDCl_3$) $\delta_C$ 17.5 ($CH_3$-3), 27.3 ($C(CH_3)_3$), 34.7 ($CH_3$-8), 47.0 (**C**H-2), 52.1 ($CH_2$-6), 66.2 ($OCH_2Ar$), 79.8 (**C**$(CH_3)_3$), 127.1-127.6 ($CH_{Ar}$), 134.3 ($CH_{Ar}$ quat.), 155.0 (**C** = O-9), 167.9 (**C** = O-5), 171.5 (**C** = O-1); HRMS (NSI) calcd for $(C_{18}H_{27}N_2O_5)^+$, $MH^+$: 351.1914, found 351.1916. LCMS purity >95% (C-18 reversed phase, MeOH-$H_2O$).

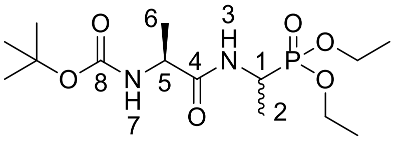


***Tert*-butyl ((2*S*)-1-((-1-(diethoxyphosphoryl)ethyl)amino)-1-oxopropan-2-yl)carbamate or Boc-L-Ala-D/L-Fos diethyl ester (19d)**. General peptide coupling method was followed, using Boc-L-Ala-OH (**15d**) (10.0 mmol, 1.90 g) in dry THF and diethyl 1-aminoethylphosphonate (**9**) (10.0 mmol, 1.84 g) in dry THF. The pale yellow crude syrup was purified by column chromatography, using 100% DCM and increasing to 95:5 DCM/methanol, to afford **19d** as an off-white solid composed of 2 diastereoisomers, Boc-L-Ala-L-Fos diethyl ester and Boc-L-Ala-D-Fos diethyl ester (2.49 g, 7.1 mmol, 71%); m.p. 102 – 105 °C; $\bar{\upsilon}_{max}$/cm$^{-1}$ 3280 (NH), 1710 (C = O), 1652 (C = O), 1556 (NH bend), 1229 (P = O), 1173 (C-O), 1013 (P-O-C), 973 (P-O-C); $^1$H NMR (300 MHz, $CDCl_3$) $\delta_H$ 1.23-1.43 (12H, m, $CH_3$-2, $CH_3$-6, 2 x $OCH_2CH_3$), 1.44 (9H, s, $C(CH_3)_3$), 4.06-4.23 (5H, m, 2 x $OCH_2CH_3$, **CH**-5), 4.40-4.52 (1H, m, **CH**-1), 5.12 (0.5H, d, $^3J_{H-H}$ = 1.5 Hz, N**H**-7), 5.14 (0.5H, d, $^3J_{H-H}$ = 1.5 Hz, N**H**-7), 6.72 (0.5H, d, $^3J_{H-H}$ = 2.3 Hz, N**H**-3), 6.74 (0.5H, d, $^3J_{H-H}$ = 2.3 Hz, N**H**-3); $^{13}$C NMR (75 MHz, $CDCl_3$) $\delta_C$ 15.6 ($CH_3$-2), 16.3 (d, $^3J_{C-P}$ = 3.0 Hz, $OCH_2CH_3$), 16.4 (d, $^3J_{C-P}$ = 2.3 Hz, $OCH_2CH_3$), 16.5 (d, $^3J_{C-P}$ = 3.0 Hz, $OCH_2CH_3$), 16.6 (d, $^3J_{C-P}$ = 2.3 Hz, $OCH_2CH_3$), 18.4 ($CH_3$-6), 28.3 ($C(CH_3)_3$), 40.8 (d, $^1J_{C-P}$ = 156.8 Hz, **C**H-1), 41.0 (d, $^1J_{C-P}$ = 156.8 Hz, **C**H-1), 50.0 (**C**H-5), 62.4 (d, $^2J_{C-P}$ = 6.8 Hz, $OCH_2CH_3$), 62.5 (d, $^2J_{C-P}$ = 6.8 Hz, $OCH_2CH_3$), 62.6 (d, $^2J_{C-P}$ = 6.8 Hz, $OCH_2CH_3$), 62.8 (d, $^2J_{C-P}$ = 6.8 Hz, $OCH_2CH_3$), 80.0 (**C**$(CH_3)_3$), 155.2 (**C** = O-8), 172.1 (**C** = O-4); $^{31}$P-$^1$H$_{decoup}$ NMR (121 MHz, $CDCl_3$) $\delta_P$ 25.2; HRMS (NSI) calcd for $(C_{17}H_{35}N_3O_7P)^+$, $MH^+$: 424.2207, found 424.2200; CHN (Found: C, 48.22; H, 8.58; N, 7.87. $C_{14}H_{29}N_2O_6P$ requires C, 47.92; H, 8.30; N, 7.95%).

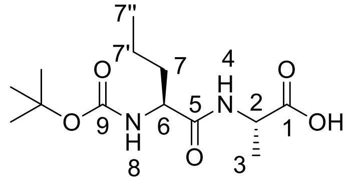


**(*S*)-2-((*S*)-2-((*Tert*-butoxycarbonyl)amino)pentanamido) propanoic acid or Boc-L-Nva-L-Ala-OH (17a)**. Deprotection of benzyl ester was followed, using (*S*)-benzyl 2-((*S*)-2-((*tert*-butoxycarbonyl)amino)pentanamido)propanoate (**16a**) (6.0 mmol, 2.27 g) to afford **17a** as a white solid (1.66 g, 5.7 mmol, 96.0%); m.p. 55 - 58 °C (decomp.); $\bar{\upsilon}_{max}$/cm$^{-1}$ 3500-3000 (br, OH), 3300 (NH), 1688 (br C = O), 1655 (C = O), 1522 (NH bend), 1245 (C-O), 1164 (C-O); $^1$H NMR (300 MHz, $CDCl_3$) $\delta_H$ 0.85 (3H, t, $^3J_{H-H}$ = 9.0 Hz, $CH_3$-7''), 1.27-1.31 (5H, m, $CH_3$-3, $CH_2$-7'), 1.39 (9H, s, $C(CH_3)_3$), 1.48-1.53 (1H, m, $CH_{a/b}$-7), 1.67-1.71 (1H, m, $CH_{a/b}$-7), 4.10 (1H, m, **CH**-6), 4.50 (1H, m, **CH**-2), 5.27 (1H, m, N**H**-8), 6.93 (1H, m, NH-4), 8.87 (1H, br, O**H**); $^{13}$C NMR (75 MHz, $CDCl_3$) $\delta_C$ 13.7 ($CH_3$-7''), 18.0 ($CH_3$-3), 18.8 ($CH_2$-7'), 28.3 ($C(CH_3)_3$), 34.5 ($CH_2$-7), 48.1 (**C**H-2), 54.3 (**C**H-6), 80.4 (**C**$(CH_3)_3$), 156.0 (**C** = O-9), 172.5 (**C** = O-5), 175.5 (**C** = O-1); HRMS (NSI) calcd for $(C_{13}H_{25}N_2O_5)^+$, $MH^+$: 289.1758, found 289.1758; CHN (Found: C, 54.18; H, 8.78; N, 9.62. $C_{13}H_{24}N_2O_5$ requires C, 54.15; H, 8.39; N, 9.72%).

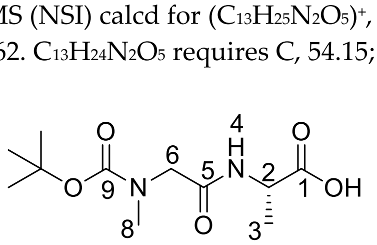

**(*S*)-Benzyl 2-(2-((*tert*-butoxycarbonyl)(methyl)amino)acetamido) propanoic acid or Boc-Sar-L-Ala-OH (17b)**. Deprotection of benzyl ester was followed, using (*S*)-benzyl 2-(2-((*tert*-butoxycarbonyl)(methyl)amino)acetamido) propanoate (**16b**) (10.0 mmol, 3.51 g) to afford **17b** as a colorless syrup (2.50 g, 9.6 mmol, 96%); $\bar{\upsilon}_{max}$/cm$^{-1}$ 3301 (NH), 2961 (broad OH), 1736 (C = O), 1664 (br C = O), 1542 (NH bend), 1241 (C-O), 1147 (C-O); $^1$H NMR (300 MHz, $CDCl_3$) $\delta_H$ 1.36 (3H, t, $^3J_{H-H}$ = 6.0 Hz, **CH**$_3$-3), 1.39 (9H, s, C(**CH**$_3$)$_3$), 2.89 (3H, s, **CH**$_3$-8), 3.72 (1H, d, $^2J_{H-H}$ = 18.0 Hz, **CH**$_{a/b}$-6), 3.98 (1H, d, $^2J_{H-H}$ = 18.0 Hz, **CH**$_{a/b}$-6), 4.57 (1H, m, **CH**-2), 6.96 (1H, m, **NH**-4), 7.26 (1H, br, **OH**); $^{13}$C NMR (75 MHz, $CDCl_3$) $\delta_C$ 17.2 (**C**H$_3$-3), 27.3 (C(**C**H$_3$)$_3$), 46.8 (**C**H-2), 49.6 (**C**H$_3$-8), 52.1 (**C**H$_2$-6), 80.6 (**C**(CH$_3$)$_3$), 155.5 (**C** = O-9), 168.3 (**C** = O-5), 174.1 (**C** = O-1); HRMS (NSI) calcd for $(C_{11}H_{19}N_2O_5)^-$, MH$^-$: 259.1299, found 259.1295. LCMS purity >95% (C-18 reversed phase, MeOH-$H_2O$).

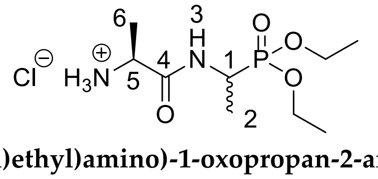


**(2*S*)-1-((1-(Diethoxyphosphoryl)ethyl)amino)-1-oxopropan-2-aminium chloride or L-Ala-D/L-Fos diethyl ester hydrochloride (20d)**. Deprotection of *tert*-butoxycarbonyl was followed, using *tert*-butyl ((2*S*)-1-((-1-(diethoxyphosphoryl)ethyl)amino)-1-oxopropan-2-yl)carbamate (**19d**) (6.0 mmol, 2.13 g). The off-white hygroscopic crude solid was washed with petrol to afford **20d** as a pale green solid composed of 2 diastereoisomers, L-Ala-L-Fos diethyl ester hydrochloride and L-Ala-D-Fos diethyl ester hydrochloride (1.46 g, 5.1 mmol, 84%); m.p. 60 – 63 °C; $\bar{\upsilon}_{max}$/cm$^{-1}$ 2986 (NH$^+$), 1673 (C = O), 1555 (NH bend), 1212(P = O), 1017 (P-O-C), 970 (P-O-C); $^1$H NMR (300 MHz, $CD_3OD$) $\delta_H$ 1.29-1.44 (9H, m, 2 x OCH$_2$**CH**$_3$, **CH**$_3$-2), 1.51 (3H, d, $^3J_{H-H}$ = 6.0 Hz, **CH**$_3$-6), 3.90-3.98 (1H, m, **CH**-5), 4.08-4.22 (4H, m, 2 x O**CH**$_2$CH$_3$), 4.28-4.47 (1H, m, **CH**-1); $^{13}$C NMR (75 MHz, $CD_3OD$) $\delta_C$ 13.7 (**C**H$_3$-2), 14.0 (**C**H$_3$-2), 15.4 (2 x OCH$_2$CH$_3$), 16.3 (**C**H$_3$-6), 41.1 (d, $^1J_{C-P}$ = 158.3 Hz, **C**H-1), 41.4 (d, $^1J_{C-P}$ = 158.3 Hz, **C**H-1), 48.8 (**C**H-5), 48.9 (**C**H-5), 62.7-63.0 (2 x OCH$_2$CH$_3$), 169.0 (**C** = O-4); $^{31}$P-$^1$H$_{decoup}$ NMR (121 MHz, $CDCl_3$) $\delta_P$ 29.0, 29.1; HRMS (NSI) calcd for $(C_9H_{22}N_2O_4P)^+$, M$^+$: 253.1312, found 253.1316. LCMS purity >95% (C-18 reversed phase, MeOH-$H_2O$).

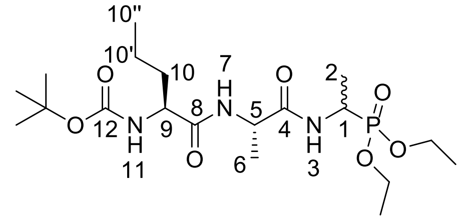


***Tert*-butyl ((2*S*)-1-(((2*S*)-1-((1-(diethoxyphosphoryl)ethyl) amino)-1-oxopropan-2-yl)amino)-1-oxopentan-2-yl)carbamate or Boc-L-Nva-L-Ala-D/L-Fos diethyl ester (18a)**. General peptide coupling method was followed, using (*S*)-2-((*S*)-2-((*tert*-butoxycarbonyl)amino)pentanamido)propanoic acid (**17a**) (5.0 mmol, 1.45 g) in dry THF and diethyl 1-aminoethylphosphonate (**9**) (4.8 mmol, 0.87 g) in dry THF. The white crude solid was purified by column chromatography using 100% DCM, increasing to 90:10 DCM/methanol, to afford **18a** as a white solid composed of 2 diastereoisomers, Boc-L-Nva-L-Ala-L-Fos diethyl ester and Boc-L-Nva-L-Ala-D-Fos diethyl ester (1.70 g, 3.8 mmol, 78%); m.p. 165 - 168 °C; $\bar{\upsilon}_{max}$/cm$^{-1}$ 3267 (NH), 1708 (C = O), 1638 (br C = O), 1537 (NH bend), 1227 (P = O), 1165 (C-O), 1019 (P-O-C), 966 (P-O-C); $^1$H NMR (300 MHz, $CDCl_3$) $\delta_H$ 0.85 (3H, t, $^3J_{H-H}$ = 9.0 Hz, **CH**$_3$-10''), 1.18-1.34 (14H, m, 2 x OCH$_2$**CH**$_3$, **CH**$_3$-2, **CH**$_3$-6, **CH**$_2$-10'), 1.37 (9H, s, C(**CH**$_3$)$_3$), 1.47-1.54 (1H, m, **CH**$_{a/b}$-10), 1.65-1.73 (1H, m, **CH**$_{a/b}$-10), 3.98-4.12 (5H, m, 2 x O**CH**$_2$CH$_3$, **CH**-9), 4.33-4.44 (1H, m, **CH**-1), 4.48-4.54 (1H, m, **CH**-5), 5.19 (0.5H, d, $^3J_{H-H}$ = 6.0 Hz, **NH**-11), 5.23 (0.5H, d, $^3J_{H-H}$ = 6.0 Hz, **NH**-11), 6.78 (0.5H, d, $^3J_{H-H}$ = 6.0 Hz, **NH**-7), 6.87 (0.5H, d, $^3J_{H-H}$ = 6.0 Hz, **NH**-7), 7.15 (0.5H, d, $^3J_{H-H}$ = 9.0 Hz, **NH**-3), 7.23 (0.5H, d, $^3J_{H-H}$ = 9.0 Hz, **NH**-3); $^{13}$C NMR (75 MHz, $CDCl_3$) $\delta_C$ 12.7 (**C**H$_3$-10''), 14.4 (**C**H$_3$-2), 14.5 (**C**H$_3$-2), 15.3 (OCH$_2$**C**H$_3$), 15.4 (OCH$_2$**C**H$_3$), 15.5 (OCH$_2$**C**H$_3$), 15.6 (OCH$_2$**C**H$_3$), 17.6 (**C**H$_3$-6), 17.7 (**C**H$_3$-6), 17.8 (**C**H$_2$-10'), 17.9 (**C**H$_2$-10'), 27.3 (C(**C**H$_3$)$_3$), 33.8 (**C**H$_2$-10), 33.9 (**C**H$_2$-10), 39.9 (d, $^1J_{P-C}$ = 157.5 Hz, **C**H-1), 40.0 (d, $^1J_{P-C}$ = 156.8 Hz, **C**H-1), 47.7 (**C**H-5), 47.9 (**C**H-5), 53.5 (**C**H-9), 53.5 (**C**H-9), 61.5 (d,

$^2J_{C-P}$ = 7.5 Hz, O**C**H$_2$CH$_3$), 61.6 (d, $^2J_{C-P}$ = 7.5 Hz, O**C**H$_2$CH$_3$), 61.7 (d, $^2J_{C-P}$ = 7.5 Hz, O**C**H$_2$CH$_3$), 61.9 (d, $^2J_{C-P}$ = 7.5 Hz, O**C**H$_2$CH$_3$), 78.9 (**C**(CH$_3$)$_3$), 154.7 (C = O-12), 170.6 (**C** = O-4 or **C** = O-8), 170.7 (**C** = O-4 or **C** = O-8), 171.0 (**C** = O-4 or **C** = O-8), 171.1 (**C** = O-4 or **C** = O-8); $^{31}$P-$^1$H$_{decoup}$ NMR (121 MHz, CDCl$_3$) $\delta_P$ 25.0, 25.1; HRMS (NSI) calcd for ($C_{19}H_{39}N_3O_7P$)$^+$, MH$^+$: 452.2520, found 452.2518; CHN (Found: C, 50.74; H, 8.55; N, 9.51. $C_{19}H_{38}N_3O_7P$ requires C, 50.54; H, 8.48; N, 9.31%).

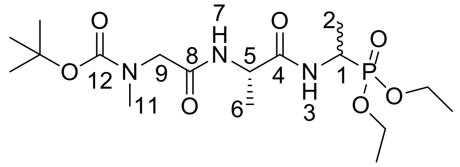


***Tert*-butyl (2-(((2*S*)-1-((1-(diethoxyphosphoryl)ethyl)amino)-1-oxopropan-2-yl)amino)-2-oxoethyl)(methyl)carbamate or Boc-Sar-L-Ala-D/L-Fos (18b)**. General peptide coupling method was followed, using (*S*)-benzyl 2-(2-((*tert*-butoxycarbonyl)(methyl)amino)acetamido)propanoic acid (**17b**) (6.0 mmol, 1.57 g) in dry THF and diethyl 1-aminoethylphosphonate (**9**) (6.0 mmol, 1.10 g) in dry THF. The yellow crude liquid was purified by column chromatography, using 100% DCM and increasing to 90:10 DCM/methanol, to afford **18b** as a colorless liquid composed of 2 diastereoisomers, Boc-Sar-L-Ala-L-Fos diethyl ester and Boc-Sar-L-Ala-D-Fos diethyl ester (1.60 g, 3.8 mmol, 63%); $\bar{\upsilon}_{max}$/cm$^{-1}$ 3270 (NH), 1700 (br C = O), 1655 (C = O), 1545 (NH bend), 1225 (P = O), 1149 (C-O), 1018 (P-O-C), 966 (P-O-C); $^1$H NMR (300 MHz, CDCl$_3$) $\delta_H$ 1.19-1.34 (12H, m, C**H**$_3$-2, C**H**$_3$-6, 2 x OCH$_2$C**H**$_3$), 1.40 (9H, s, C(C**H**$_3$)$_3$), 2.87 (3H, s, C**H**$_3$-11), 3.72 (0.5H, d, $^2J_{H-H}$ = 15.0 Hz, C**H**$_{a/b}$-9), 3.78 (0.5H, d, $^2J_{H-H}$ = 15.0 Hz, C**H**$_{a/b}$-9), 3.81 (0.5H, d, $^2J_{H-H}$ = 15.0 Hz, C**H**$_{a/b}$-9), 3.87 (0.5H, d, $^2J_{H-H}$ = 15.0 Hz, C**H**$_{a/b}$-9), 4.00-4.11 (4H, m, 2 x OC**H**$_2$CH$_3$), 4.35-4.43 (1H, m, C**H**-1), 4.47-4.52 (1H, m, C**H**-5), 6.67 (1H, d, $^3J_{H-H}$ = 9.0 Hz, N**H**-7), 6.98 (0.5H, d, $^3J_{H-H}$ = 9.0 Hz, N**H**-3), 7.15 (0.5H, d, $^3J_{H-H}$ = 9.0 Hz, N**H**-3); $^{13}$C NMR (75 MHz, CDCl$_3$) $\delta_C$ 15.5 (**C**H$_3$-2), 15.5 (**C**H$_3$-2), 16.3 (d, $^3J_{P-C}$ = 3.0 Hz, OCH$_2$**C**H$_3$), 16.4 (d, $^3J_{P-C}$ = 3.0 Hz, OCH$_2$**C**H$_3$), 18.7 (**C**H$_3$-6), 28.3 (C(**C**H$_3$)$_3$), 35.8 (**C**H$_3$-11), 41.0 (d, $^1J_{P-C}$ = 157.5 Hz, **C**H-1), 48.5 (**C**H-5), 53.0 (**C**H$_2$-9), 62.5 (d, $^2J_{P-C}$ = 6.8 Hz, O**C**H$_2$CH$_3$), 62.6 (d, $^2J_{P-C}$ = 6.8 Hz, O**C**H$_2$CH$_3$), 62.7 (d, $^2J_{P-C}$ = 6.8 Hz, O**C**H$_2$CH$_3$), 62.9 (d, $^2J_{P-C}$ = 6.8 Hz, O**C**H$_2$CH$_3$), 80.7 (**C**(CH$_3$)$_3$), 156.0 (C = O-12), 171.5 (**C** = O-4 or **C** = O-8), 171.6 (**C** = O-4 or **C** = O-8); $^{31}$P-$^1$H$_{decoup}$ NMR (121 MHz, CDCl$_3$) $\delta_P$ 25.0, 25.1; HRMS (NSI) calcd for ($C_{17}H_{35}N_3O_7P$)$^+$, MH$^+$: 424.2207, found 424.2203. LCMS purity >95% (C-18 reversed phase, MeOH-H$_2$O).

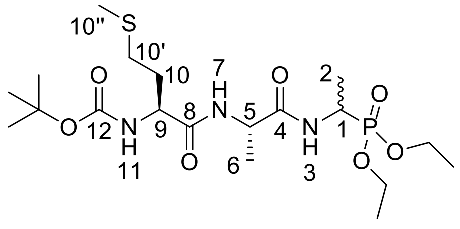


***Tert*-butyl ((2*S*)-1-(((2*S*)-1-((1-(diethoxyphosphoryl)ethyl) amino)-1-oxopropan-2-yl)amino-4-(methylthio)-1-oxobutan-2-yl)carbamate or Boc-L-Met-L-Ala-D/L-Fos diethyl ester (18c)**. General peptide coupling method was followed, using Boc-L-Met-OH (**15c**) (3.4 mmol, 0.88 g) in dry THF and (2*S*)-1-((1-(diethoxyphosphoryl)ethyl)amino)-1-oxopropan-2-aminium chloride (**20d**) (3.4 mmol, 0.97 g) in dry DCM. The yellow crude solid was purified by column chromatography (DCM/MeOH (95:5)) to give **18c** as an off-white solid composed of 2 diastereoisomers, Boc-L-Met-L-Ala-L-Fos diethyl ester and Boc-L-Met-L-Ala-D-Fos diethyl ester (0.53 g, 1.1 mmol, 32%); m.p. 172 – 176 °C; $\bar{\upsilon}_{max}$/cm$^{-1}$ 3272 (NH), 1708 (C = O), 1673 (C = O), 1637 (C = O), 1530 (NH bend), 1226 (P = O), 1165 (C-O), 1020 (P-O-C), 976 (P-O-C); $^1$H NMR (300 MHz, CDCl$_3$) $\delta_H$ 1.16-1.36 (12H, m, C**H**$_3$-2, C**H**$_3$-6, 2 x OCH$_2$C**H**$_3$), 1.36 (9H, s, C(C**H**$_3$)$_3$), 1.82-2.01 (2H, m, C**H**$_2$-10), 2.04 (3H, s, C**H**$_3$-10''), 2.49 (2H, dd, $^3J_{H-H}$ = 9.0 Hz, 3.0 Hz, C**H**$_2$-10'), 4.00-4.12 (4H, m, 2 x OC**H**$_2$CH$_3$), 4.16-4.26 (1H, m, C**H**-9), 4.33-4.43 (1H, m, C**H**-1), 4.45-4.53 (1H, m, C**H**-5), 5.40 (0.5H, d, $^3J_{H-H}$ = 9.0 Hz, N**H**-11), 5.44 (0.5H, d, $^3J_{H-H}$ = 6.0 Hz, N**H**-11), 6.85 (0.5H, d, $^3J_{H-H}$ = 6.0 Hz, N**H**-7), 6.92 (0.5H, d, $^3J_{H-H}$ = 6.0 Hz, N**H**-7), 7.07 (0.5H, d, $^3J_{H-H}$ = 9.0 Hz, N**H**-3), 7.16 (0.5H, d, $^3J_{H-H}$ = 9.0 Hz, N**H**-3); $^{13}$C NMR (75 MHz, CDCl$_3$) $\delta_C$ 14.2 (**C**H$_3$-2), 14.3 (**C**H$_3$-2), 14.5 (**C**H$_3$-10''), 14.6 (**C**H$_3$-10''), 15.4 (d, $^3J_{C-P}$ = 3.0 Hz, OCH$_2$**C**H$_3$), 15.4 (d, $^3J_{C-P}$ = 2.3 Hz, OCH$_2$**C**H$_3$), 15.5 (d, $^3J_{C-P}$ = 3.0 Hz, OCH$_2$**C**H$_3$), 15.5 (d, $^3J_{C-P}$ = 2.3 Hz, OCH$_2$**C**H$_3$), 17.7 (**C**H$_3$-6), 27.3 (C(**C**H$_3$)$_3$), 29.2 (**C**H$_2$-10'), 29.3 (**C**H$_2$-

10′), 30.8 ($CH_2$-10), 30.9 ($CH_2$-10), 39.9 (d, $^1J_{C-P}$ = 156.8 Hz, **C**H-1), 40.0 (d, $^1J_{C-P}$ = 156.8 Hz, **C**H-1), 47.9 (**C**H-5), 48.0 (**C**H-5), 52.6 (**C**H-9), 61.5 (d, $^2J_{C-P}$ = 6.8 Hz, O**C**$H_2CH_3$), 61.6 (d, $^2J_{C-P}$ = 6.8 Hz, O**C**$H_2CH_3$), 61.7 (d, $^2J_{C-P}$ = 6.8 Hz, O**C**$H_2CH_3$), 61.9 (d, $^2J_{C-P}$ = 6.8 Hz, O**C**$H_2CH_3$), 79.1 (**C**$(CH_3)_3$), 154.6 (**C** = O-12), 170.3 (**C** = O-4 or **C** = O-8), 170.4 (**C** = O-4 or **C** = O-8), 170.5 (**C** = O-4 or **C** = O-8), 170.6 (**C** = O-4 or **C** = O-8); $^{31}P$-$^1H_{decoup}$ NMR (121 MHz, $CDCl_3$) $\delta_P$ 25.0, 25.1; HRMS (NSI) calcd for $(C_{19}H_{39}N_3O_7PS)^+$, $MH^+$: 484.2241, found 484.2228. LCMS purity >95% (C-18 reversed phase, MeOH-$H_2O$).

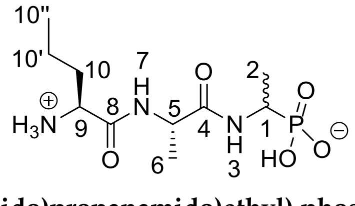

**(1-((*S*)-2-((*S*)-2-Aminopentanamido)propanamido)ethyl) phosphonic acid or L-Nva-L-Ala-D/L-Fos (21a)**. The *tert*-butoxycarbonyl and diethyl ester protecting groups of *tert*-butyl ((2*S*)-1-(((2*S*)-1-((1-(diethoxyphosphoryl)ethyl)amino)-1-oxopropan-2-yl)amino)-1-oxopentan-2-yl)carbamate (**18a**) (1.6 mmol, 0.72 g) were removed. The pale green crude solid was recrystallised from hot water/acetone to give **21a** as a pale green solid composed of 2 diastereoisomers, L-Nva-L-Ala-L-Fos and L-Nva-L-Ala-D-Fos (0.22 g, 0.75 mmol, 47%); m.p. 207 – 210 °C (decomp.); $\bar{\upsilon}_{max}$/cm$^{-1}$ 3280 ($NH^+$), 3500-2900 (br OH), 1643 (br C = O), 1552 (NH bend), 1149 (P = O), 1037 (P-O-C), 922 (P-OH); $^1H$ NMR (300 MHz, $D_2O$) $\delta_H$ 0.96 (3H, t, $^3J_{H-H}$ = 7.1 Hz, $CH_3$-10′′), 1.27-1.32 (3H, d, $^3J_{H-H}$ = 6.8 Hz, C**H**$_3$-2), 1.40-1.42 (3H, m, C**H**$_3$-6), 1.40-1.42 (2H, m, C**H**$_2$-10′), 1.88-1.86 (2H, m, C**H**$_2$-10), 4.00-4.02 (2H, m, C**H**-1, C**H**-9), 4.34-4.39 (1H, m, C**H**-5); $^{13}C$ NMR (75 MHz, $D_2O$) $\delta_C$ 13.4 (**C**$H_3$-10′′), 16.0 (**C**$H_3$-2), 17.1 (**C**$H_3$-6), 17.2 (**C**$H_3$-6), 18.1 (**C**$H_2$-10′), 18.2 (**C**$H_2$-10′), 33.5 (**C**$H_2$-10), 33.6 (**C**$H_2$-10), 50.5 (**C**H-5), 50.8 (**C**H-5), 53.5 (**C**H-1, **C**H-9), 170.4 (**C** = O-8), 170.6 (**C** = O-8),174.7 (**C** = O-4); $^{31}P$-$^1H_{decoup}$ NMR (121 MHz, $CDCl_3$) $\delta_P$ 18.5; HRMS (NSI) calcd for $(C_{10}H_{23}N_3O_5P)^+$, $MH^+$: 296.1370, found 296.1373. LCMS purity >95% (C-18 reversed phase, MeOH-$H_2O$).

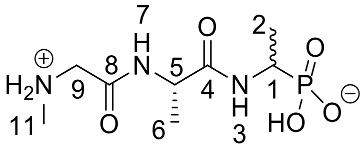

**(1-((*S*)-2-(2-(Methylamino)acetamido)propanamido)ethyl) phosphonic acid or Sar-L-Ala-D/L-Fos (21b)**. The *tert*-butoxycarbonyl and diethyl ester protecting groups of (1-((*S*)-2-((*S*)-2-aminopropanamido)propanamido)ethyl)phosphonic acid (**18b**) (3.3 mmol, 1.40 g) were removed. The pale green crude solid was recrystallised from hot water/ethanol to give **21b** as a pale green solid composed of 2 diastereoisomers, Sar-L-Ala-L-Fos and Sar-L-Ala-D-Fos (0.45 g, 1.7 mmol, 51%); m.p. 241 – 245 °C (decomp.); $\bar{\upsilon}_{max}$/cm$^{-1}$ 3289 ($NH^+$), 3500-2900 (br OH), 1632 (br C = O), 1556 (NH bend), 1174 (P = O), 1059 (P-O-C), 919 (P-OH); $^1H$ NMR (300 MHz, $D_2O$) $\delta_H$ 1.14-1.57 (6H, m, C**H**$_3$-2, C**H**$_3$-6), 2.74 (3H, s, C**H**$_3$-11), 3.84-4.07 (3H, m, C**H**$_2$-9, C**H**-1), 4.32-4.58 (1H, m, C**H**-5); $^{13}C$ NMR (75 MHz, $D_2O$) $\delta_C$ 15.4 (**C**$H_3$-2), 16.8 (**C**$H_3$-6), 32.9 (**C**$H_3$-11), 43.9 (d, $^1J_{P-C}$ = 148.5 Hz, **C**H-1), 49.4 (**C**$H_2$-9), 50.0 (**C**H-5), 166.0 (**C** = O-8), 173.7 (**C** = O-4); $^{31}P$-$^1H_{decoup}$ NMR (121 MHz, $CDCl_3$) $\delta_P$ 19.2; HRMS (NSI) calcd for $(C_8H_{19}N_3O_5P)^+$, $MH^+$: 268.1057, found 268.1016; LCMS purity >95% (C-18 reversed phase, MeOH-$H_2O$).

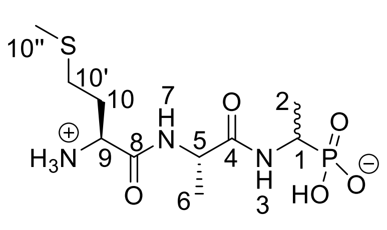

**(1-((*S*)-2-((*S*)-2-Amino-4-(methylthio)butanamido) propanamido)ethyl)phosphonic acid or L-Met-L-Ala-D/L-Fos (21c)**. The *tert*-butoxycarbonyl and diethyl ester protecting groups of *tert*-butyl ((2*S*)-1-(((2*S*)-1-((1-(diethoxyphosphoryl)ethyl)amino)-1-oxopropan-2-yl)amino-4-(methylthio)-1-

oxobutan-2-yl)carbamate (**18c**) (0.9 mmol, 0.43 g) were removed. The green crude solid was recrystallised from hot water/ethanol to give **21c** as a pale green solid composed of 2 diastereoisomers, L-Met-L-Ala-L-Fos and L-Met-L-Ala-D-Fos (0.13 g, 0.41 mmol, 46%); m.p. 214 – 217 °C (decomp.); $\bar{\upsilon}_{max}$/cm$^{-1}$ 3263 ($NH^+$), 2834 (broad OH), 1641 (br C = O), 1552 (NH bend), 1150 (P = O), 1041 (P-O-C), 919 (P-OH); $^1$H NMR (300 MHz, $D_2O$) $\delta_H$ 1.29-1.33 (3H, m, C**H**$_3$-2), 1.42 (3H, d, $^3J_{H-H}$ = 6.0 Hz, C**H**$_3$-6), 2.15 (3H, s, C**H**$_3$-10′′), 2.20 (2H, m, C**H**$_2$-10), 2.62 (2H, m, C**H**$_2$-10′), 4.05 (1H, m, C**H**-1), 4.14 (1H, m, C**H**-9), 4.39-4.41 (1H, m, C**H**-5); $^{13}$C NMR (75 MHz, $D_2O$) $\delta_C$ 16.9 (**C**H$_3$-10′′), 18.4 (**C**H$_3$-2), 19.5 (**C**H$_3$-6), 19.6 (**C**H$_3$-6), 32.8 (**C**H$_2$-10′), 33.0 (**C**H$_2$-10′), 30.7 (**C**H$_2$-10), 31.0 (**C**H$_2$-10), 44.4 (**C**H-1), 52.9 (**C**H-5), 53.0 (**C**H-5), 55.0 (**C**H-9), 176.1 (**C** = O-4, **C** = O-8); $^{31}$P-$^1$H$_{decoup}$ NMR (121 MHz, $CDCl_3$) $\delta_P$ 20.7; HRMS (NSI) calcd for $(C_{10}H_{23}N_3O_5PS)^+$, $MH^+$: 328.1091, found 328.1094; LCMS purity >95% (C-18 reversed phase, MeOH-$H_2O$).

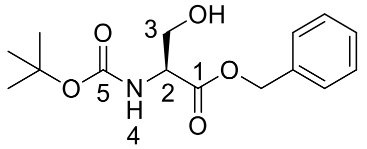


**(*S*)-Benzyl 2-((*tert*-butoxycarbonyl)amino)-3-hydroxy propanoate or Boc-L-Ser-OBzl (11)**. Boc-L-Serine (**10**) (20 mmol, 4.10 g) and 1,8-diazabicyclo[5.4.0]undec-7-ene (DBU) (30 mmol, 4.5 mL) were dissolved in a round-bottom flask containing dry benzene (80 mL), followed by the addition of benzyl bromide (30 mmol, 3.60 mL). The solution was stirred overnight at room temperature under nitrogen and later the solvent was removed under reduced pressure to afford an off-white residue. Ethyl acetate (100 mL) was added, the flask contents were sonicated and then washed with 1M HCl (50 mL) and brine (2 x 50 mL). The organic layer was dried over $MgSO_4$, filtered, concentrated *in vacuo* and purified by column chromatography (petrol/ethyl acetate (1:1)) to give **11** as a white solid (5.24 g, 17.7 mmol, 89%); m.p. 61 – 63 °C (lit. m.p. 59 – 60 °C); $\bar{\upsilon}_{max}$/cm$^{-1}$ 3416 (NH, OH), 1756 (C = O), 1666 (C = O), 1522 (NH bend), 1200 (C-O), 1154 (C-O); $^1$H NMR (300 MHz, $CDCl_3$) $\delta_H$ 1.36 (9H, s, C(C**H**$_3$)$_3$), 2.17 (1H, br, O**H**), 3.82 (1H, dd, $^2J_{H-H}$ = 11.1 Hz, $^3J_{H-H}$ = 3.6 Hz, C**H**$_{a/b}$-3), 3.90 (1H, dd, $^2J_{H-H}$ = 11.1 Hz, $^3J_{H-H}$ = 3.9 Hz, C**H**$_{a/b}$-3), 4.33 (1H, m, C**H**-2), 5.11 (1H, d, $^2J_{H-H}$ = 12.3 Hz, OC**H**$_{a/b}$Ar), 5.16 (1H, d, $^2J_{H-H}$ = 12.3 Hz, OC**H**$_{a/b}$Ar), 5.40 (1H, br, N**H**-4), 7.27 (5H, m, 5 x C**H**$_{Ar}$); $^{13}$C NMR (75 MHz, $CDCl_3$) $\delta_C$ 27.1 (C(**C**H$_3$)$_3$), 54.7 (**C**H-2), 62.3 (**C**H$_2$-3), 66.2 (O**C**H$_2$Ar), 79.1 (**C**(CH$_3$)$_3$), 127.0 (2 x **C**H$_{Ar}$), 127.3 (**C**H$_{Ar}$), 127.4 (2 x **C**H$_{Ar}$), 134.1 (**C**H$_{Ar}$ quat.), 153.0 (**C** = O-5), 170.7 (**C** = O-1); *m/z* (ESI) calcd for $(C_{15}H_{21}NNaO_5)^+$, $MNa^+$: 318.3, found 318.2.

lit. m.p.; Lavielle, S.; Ling, N.C.; Saltman, R.; Guillemn, R.C. *Carbohydr. Res*. **1981**, 89, 229-236. (DOI: 10.1016/s0008-6215(00)85248-9)

Benzene is a known carcinogen and extra safety measure where PPEs were used at all time throughout the synthesis and experiments were only performed in the certified fume cupboard.

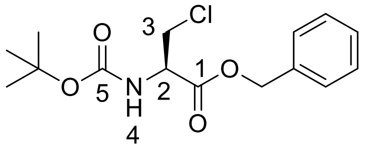


**(*R*)-Benzyl 2-((*tert*-butoxycarbonyl)amino)-3-chloropropanoate or Boc-β-Cl-L-Ala-OBzl (12)**. (*S*)-Benzyl 2-((*tert*-butoxycarbonyl)amino)-3-hydroxypropanoate (**11**) (15 mmol, 4.43 g) was dissolved in dry DCM (40 mL), followed by the addition of trichloroacetonitrile (30 mmol, 3 ml). The solution was stirred at room temperature for 2 hours. To this solution, triphenylphosphine (30 mmol, 7.87 g) in dry DCM (50 mL) was added slowly. The resulting solution was stirred overnight at room temperature under nitrogen; brine (100 mL) was added to quench the reaction. The organic layer was washed with brine (3 x 100 mL), dried over $MgSO_4$, filtered and concentrated in vacuo to afford an orange residue. The residue was purified by column chromatography (petrol/ethyl acetate (7:3)) to give **12** as a white solid (3.53 g, 11.2 mmol, 75%); m.p. 55 – 58 °C; $\bar{\upsilon}_{max}$/cm$^{-1}$ 3364 (NH), 1725 (C = O), 1680 (C = O), 1519 (NH bend), 1208 (C-O), 1158 (C-O); $^1$H NMR (300 MHz, $CDCl_3$) $\delta_H$ 1.38 (9H, s, C(C**H**$_3$)$_3$), 3.78 (1H, dd, $^2J_{H-H}$ = 11.2 Hz, $^3J_{H-H}$ = 3.2 Hz, C**H**$_{a/b}$-3), 3.92 (1H, dd, $^2J_{H-H}$ = 11.3 Hz, $^3J_{H-H}$ = 3.0

Hz, $CH_{a/b}$-3), 4.67 (1H, m, CH-2), 5.13 (1H, d, $^2J_{H-H}$ = 12.2 Hz, $OCH_{a/b}Ar$), 5.18 (1H, d, $^2J_{H-H}$ = 12.2 Hz, $OCH_{a/b}Ar$), 5.37 (1H, d, $^3J_{H-H}$ = 7.5 Hz, NH-4), 7.29 (5H, m, 5 x $CH_{Ar}$); $^{13}$C NMR (75 MHz, $CDCl_3$) $\delta_C$ 28.3 (C($CH_3$)$_3$), 45.5 ($CH_2$-3), 54.5 (CH-2), 67.8 ($OCH_2Ar$), 80.5 (C($CH_3$)$_3$), 128.4 ($CH_{Ar}$), 128.6 ($CH_{Ar}$), 128.7 ($CH_{Ar}$), 134.9 ($CH_{Ar}$ quat.), 155.0 (C = O-5), 169.0 (C = O-1); *m/z* (ESI) calcd for $(C_{15}H_{20}ClNNaO_4)^+$, $MNa^+$: 336.1 ($^{35}$Cl), 338.1 ($^{37}$Cl), found 336.2 ($^{35}$Cl), 338.2 ($^{37}$Cl); CHN (Found: C, 57.71; H, 6.46; N, 4.38. $C_{15}H_{20}ClNO_4$ requires C, 57.42; H, 6.42; N, 4.46%).

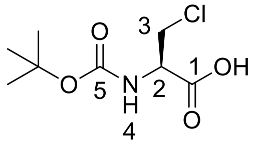


**(*R*)-2-((*Tert*-butoxycarbonyl)amino)-3-chloropropanoic acid or Boc-β-Cl-L-Ala-OH (13)**. Deprotection of benzyl ester was followed, using (*R*)-benzyl 2-((*tert*-butoxycarbonyl)amino)-3-chloropropanoate (**12**) (7.0 mmol, 2.20 g) to afford **13** as an off-white solid (1.52 g, 6.78 mmol, 97%); m.p. 125 – 128 °C (lit. m.p. 127 – 129 °C); $\bar{\nu}_{max}$/cm$^{-1}$ 3434 (NH), 2973 (br OH), 1752 (C = O), 1735 (C = O), 1519 (NH bend), 1159 (C-O), 1148 (C-O); $^1$H NMR (300 MHz, $CDCl_3$) $\delta_H$ 1.40 (9H, s, C($CH_3$)$_3$), 3.80 (1H, dd, $^2J_{H-H}$ = 12.0 Hz, $^3J_{H-H}$ = 3.0 Hz, $CH_{a/b}$-3), 3.95 (1H, dd, $^2J_{H-H}$ = 12.0 Hz, $^3J_{H-H}$ = 3.0 Hz, $CH_{a/b}$-3), 4.70 (1H, m, CH-2), 5.42 (1H, d, $^3J_{H-H}$ = 7.2 Hz, NH-4), 9.03 (1H, br, OH); $^{13}$C NMR (75 MHz, $CDCl_3$) $\delta_C$ 27.1 (C($CH_3$)$_3$), 44.0 ($CH_2$-3), 53.1 (CH-2), 79.8 (C($CH_3$)$_3$), 154.2 (C = O-5), 172.1 (C = O-1); *m/z* (ESI) calcd for $(C_8H_{14}ClNNaO_4)^+$, $MNa^+$: 246.1 ($^{35}$Cl), 248.1 ($^{37}$Cl), found 246.1 ($^{35}$Cl), 248.1 ($^{37}$Cl).
lit. m.p.; Cheung, K.S.; Wasserman, S.A.; Dudek, E.; Lerner, S.A.; Johnston, M. *J. Med. Chem.* **1983**, 26, 1733-1741 (DOI: 10.1021/jm00366a015).

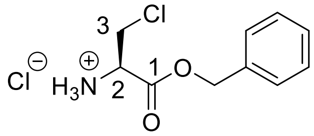


**(*R*)-1-(Benzyloxy)-3-chloro-1oxopropan-2-aminium chloride or β-Cl-L-Ala-OBzl hydrochloride (14)**. Deprotection of *tert*-butoxycarbonyl was followed, using (*R*)-benzyl 2-((*tert*-butoxycarbonyl)amino)-3-chloropropanoate (**12**) (15 mmol, 4.71 g). The white crude solid was filtered and washed by diethyl ether to give **14** as a white solid (3.47 g, 13.4 mmol, 93%); m.p. 145 °C (sub); $\bar{\nu}_{max}$/cm$^{-1}$ 2841 ($NH^+$), 1750 (C = O), 1231 (C-O); $^1$H NMR (300 MHz, $D_2O$) $\delta_H$ 4.06 (1H, dd, $^2J_{H-H}$ = 15.0 Hz, $^3J_{H-H}$ = 6.0 Hz, $CH_{a/b}$-3), 4.20 (1H, dd, $^2J_{H-H}$ = 15.0 Hz, $^3J_{H-H}$ = 6.0 Hz, $CH_{a/b}$-3), 4.70 (1H, t, $^3J_{H-H}$ = 6.0 Hz, CH-2), 5.29 (1H, d, $^2J_{H-H}$ = 12.0 Hz, $OCH_{a/b}Ar$), 5.37 (1H, d, $^2J_{H-H}$ = 12.0 Hz, $OCH_{a/b}Ar$), 7.42-7.47 (5H, m, 5 x $CH_{Ar}$); $^{13}$C NMR (75 MHz, $D_2O$) $\delta_C$ 41.8 ($CH_2$-3), 54.0 (CH-2), 69.1 ($OCH_2Ar$), 128.6-129.1 ($CH_{Ar}$), 134.5 ($CH_{Ar}$ quart.), 167.0 (C = O-1); *m/z* (ESI) calcd for $(C_{10}H_{13}ClNO_2)$, $M^+$: 214.1 ($^{35}$Cl), 216.1 ($^{37}$Cl), found 214.1 ($^{35}$Cl), 216.1 ($^{37}$Cl); CHN (Found: C, 47.16; H, 5.43; N, 5.43. $C_{10}H_{13}Cl_2NO_2 \cdot 0.2H_2O$ requires C, 47.34; H, 5.32; N, 5.52%).

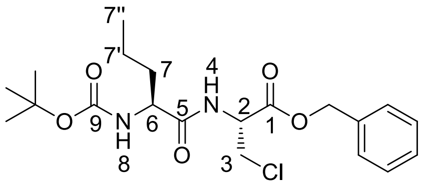


**(*R*)-Benzyl 2-((*S*)-2-((tert-butoxycarbonyl)amino)pentanamido)-3-chloropropanoate or Boc-L-Nva-β-chloro-L-Ala-OBzl (22a)**. General peptide coupling method was followed, using Boc-L-Nva-OH (**15a**) (6.0 mmol, 1.31 g) in dry THF and (*R*)-1-(benzyloxy)-3-chloro-1oxopropan-2-aminium chloride (**14**) (5.4 mmol, 1.36 g) in dry DCM. The yellow crude liquid was purified by column chromatography (40-60 petrol/ethyl acetate (7:3)) to give **22a** as a white solid (1.74 g, 4.2 mmol, 78%); m.p. 95 – 98 °C; $\bar{\nu}_{max}$/cm$^{-1}$ 3327 (NH), 1743 (C = O), 1688 (C = O), 1653 (C = O), 1518 (NH bend), 1206 (C-O), 1169 (C-O); $^1$H NMR (300 MHz, $CDCl_3$) $\delta_H$ 0.92 (3H, t, $^3J_{H-H}$ = 9.0 Hz, $CH_3$-7''), 1.32-1.43 (2H, m, $CH_2$-7'), 1.45 (9H, s, C($CH_3$)$_3$), 1.52-1.65 (1H, m, $CH_{a/b}$-7), 1.75-1.82 (1H, m, $CH_{a/b}$-7), 3.89 (1H, dd, $^2J_{H-H}$

= 12.0 Hz, $^3J_{H\text{-}H}$ = 3.0 Hz, C**H**$_{a/b}$-3), 3.99 (1H, dd, $^2J_{H\text{-}H}$ = 12.0 Hz, $^3J_{H\text{-}H}$ = 3.0 Hz, C**H**$_{a/b}$-3), 4.11-4.15 (1H, m, C**H**-6), 4.96-5.00 (2H, m, C**H**-2, N**H**-8), 5.20 (1H, d, $^2J_{H\text{-}H}$ = 12.0 Hz, OC**H**$_{a/b}$Ar), 5.25 (1H, d, $^2J_{H\text{-}H}$ = 12.0 Hz, OC**H**$_{a/b}$Ar), 6.97 (1H, d, $^3J_{H\text{-}H}$ = 6.0 Hz, N**H**-4), 7.33-7.37 (5H, m, 5 x C**H**$_{Ar}$); $^{13}$C NMR (75 MHz, $CDCl_3$) $\delta_C$ 12.7 (**C**$H_3$-7''), 17.8 (**C**$H_2$-7'), 27.3 (C(**C**$H_3$)$_3$), 33.4 (**C**$H_2$-7), 43.8 (**C**$H_2$-3), 52.2 (**C**H-2), 53.4 (**C**H-6), 67.0 (O**C**$H_2$Ar), 79.3 (**C**($CH_3$)$_3$), 127.4 (**C**H$_{Ar}$), 127.6 (**C**H$_{Ar}$), 127.7 (**C**H$_{Ar}$), 133.8 (**C**H$_{Ar}$ quat.), 154.5 (**C** = O-9), 167.5 (**C** = O-1), 171.2 (**C** = O-5); HRMS (NSI) calcd for ($C_{20}H_{30}ClN_2O_5$)$^+$, MH$^+$: 413.1838 ($^{35}$Cl), 415.1809 ($^{37}$Cl), found 413.1837 ($^{35}$Cl), 415.1807 ($^{37}$Cl); CHN (Found: C, 58.49; H, 7.22; N, 6.81. $C_{20}H_{29}ClN_2O_5$ requires C, 58.18; H, 7.08; N, 6.78%).

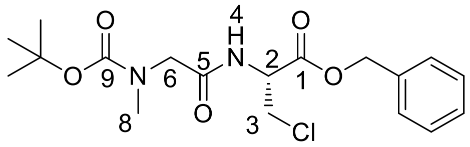

**(*R*)-Benzyl 2-(2-((*tert*-butoxycarbonyl)(methyl)amino) acetamido)-3-chloropropanoate or Boc-Sar-β-chloro-L-Ala-OBzl (22b)**. General peptide coupling method was followed, using Boc-Sar-OH (**15b**) (13.0 mmol, 2.46 g) in dry THF and (*R*)-1-(benzyloxy)-3-chloro-1oxopropan-2-aminium chloride (**14**) (13.3 mmol, 3.34 g) in dry DCM. The yellow crude liquid was purified by column chromatography (40-60 petrol/ethyl acetate (7:3)) to give **22b** as a light yellow syrup (3.63 g, 9.4 mmol, 73%); $\bar{\upsilon}_{max}$/cm$^{-1}$ 3302 (NH), 1747 (C = O), 1686 (br C = O), 1522 (NH bend), 1175 (C-O), 1148 (C-O); $^1$H NMR (300 MHz, $CDCl_3$) $\delta_H$ 1.40 (9H, s, C(C**H**$_3$)$_3$), 2.87 (3H, s, NC**H**$_3$-8), 3.80 (1H, d, $^2J_{H\text{-}H}$ = 15.0 Hz, C**H**$_{a/b}$-6), 3.82 (1H, d, $^2J_{H\text{-}H}$ = 15.0 Hz, C**H**$_{a/b}$-6), 3.83 (1H, dd, $^2J_{H\text{-}H}$ = 12.0 Hz, $^3J_{H\text{-}H}$ = 3.0 Hz, C**H**$_{a/b}$-3), 3.94 (1H, dd, $^2J_{H\text{-}H}$ = 12.0 Hz, $^3J_{H\text{-}H}$ = 3.0 Hz, C**H**$_{a/b}$-3), 4.91-4.96 (1H, m, C**H**-2), 5.13 (1H, d, $^2J_{H\text{-}H}$ = 12.0 Hz, OC**H**$_{a/b}$Ar), 5.18 (1H, d, $^2J_{H\text{-}H}$ = 12.0 Hz, OC**H**$_{a/b}$Ar), 6.97 (1H, d, $^3J_{H\text{-}H}$ = 6.0 Hz, N**H**-4), 7.26-7.30 (5H, m, 5 x C**H**$_{Ar}$); $^{13}$C NMR (75 MHz, $CDCl_3$) $\delta_C$ 28.2 (C(**C**$H_3$)$_3$), 35.6 (N**C**$H_3$-8), 44.9 (**C**$H_2$-3), 53.0 (**C**$H_2$-6), 53.0 (**C**H-2), 68.0 (O**C**$H_2$Ar), 81.0 (**C**($CH_3$)$_3$), 128.4 (**C**H$_{Ar}$), 128.6 (**C**H$_{Ar}$), 128.7 (**C**H$_{Ar}$), 134.8 (**C**H$_{Ar}$ quat.), 154.5 (**C** = O-9), 168.4 (**C** = O-1), 169.4 (**C** = O-5); HRMS (NSI) calcd for ($C_{18}H_{26}ClN_2O_5$)$^+$, MH$^+$: 385.1525 ($^{35}$Cl), 387.1496 ($^{37}$Cl), found 385.1527 ($^{35}$Cl), 387.1498 ($^{37}$Cl). LCMS purity >95% (C-18 reversed phase, MeOH-$H_2O$).

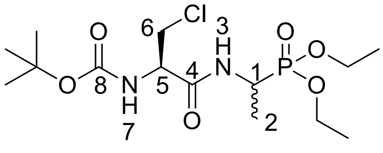

***Tert*-butyl ((2*R*)-3-chloro-1-((1-(diethoxyphosphoryl)ethyl) amino)-1-oxopropan-2-yl)carbamate or Boc-β-chloro-L-Ala-D/L-Fos diethyl ester (19e)**. General peptide coupling method was followed, using (*R*)-2-((*tert*-butoxycarbonyl)amino)-3-chloropropanoic acid (**13**) (6.0 mmol, 1.34 g) in dry THF and diethyl 1-aminoethylphosphonate (**9**) (6.0 mmol, 1.09 g) in dry THF. The light yellow crude liquid was purified by column chromatography, using 100% petrol and increasing to 100% ethyl acetate, to afford **19e** as colorless syrup composed of 2 diastereoisomers, Boc-β-Cl-L-Ala-L-Fos diethyl ester and Boc-β-Cl-L-Ala-D-Fos diethyl ester (2.03 g, 5.2 mmol, 88%); $\bar{\upsilon}_{max}$/cm$^{-1}$ 3261 (NH), 1713 (C = O), 1670 (C = O), 1517 (NH bend), 1225 (P = O), 1164 (C-O), 1020 (P-O-C), 970 (P-O-C); $^1$H NMR (300 MHz, $CDCl_3$) $\delta_H$ 1.15-1.46 (9H, m, C**H**$_3$-2, 2 x OCH$_2$C**H**$_3$), 1.47 (9H, s, C(C**H**$_3$)$_3$), 3.74 (1H, dd, $^2J_{H\text{-}H}$ = 12.0 Hz, $^3J_{H\text{-}H}$ = 6.0 Hz, C**H**$_{a/b}$-6), 4.00 (1H, dd, $^2J_{H\text{-}H}$ = 12.0 Hz, $^3J_{H\text{-}H}$ = 6.0 Hz, C**H**$_{a/b}$-6), 4.06-4.22 (4H, m, 2 x OC**H**$_2$CH$_3$), 4.40-4.56 (2H, m, C**H**-1, C**H**-5), 5.46 (0.5H, d, $^3J_{H\text{-}H}$ = 6.0 Hz, N**H**-3 or N**H**-7), 5.48 (0.5H, d, $^3J_{H\text{-}H}$ = 9.0 Hz, N**H**-3 or N**H**-7), 7.01 (0.5H, m, N**H**-3 or N**H**-7), 7.09 (0.5H, m, N**H**-3 or N**H**-7); $^{13}$C NMR (75 MHz, $CDCl_3$) $\delta_C$ 15.6 (**C**$H_3$-2), 15.7 (**C**$H_3$-2), 16.3 (d, $^3J_{C\text{-}P}$ = 1.5 Hz, OCH$_2$**C**H$_3$), 16.4 (d, $^3J_{C\text{-}P}$ = 2.3 Hz, OCH$_2$CH$_3$), 16.4 (d, $^3J_{C\text{-}P}$ = 1.5 Hz, OCH$_2$**C**H$_3$), 16.4 (d, $^3J_{C\text{-}P}$ = 2.3 Hz, OCH$_2$**C**H$_3$), 28.2 (C(**C**$H_3$)$_3$), 41.2 (d, $^1J_{C\text{-}P}$ = 157.5 Hz, **C**H-1), 41.3 (d, $^1J_{C\text{-}P}$ = 157.5 Hz, **C**H-1), 55.2 (**C**$H_2$-6), 55.3 (**C**H-5), 62.6 (d, $^2J_{C\text{-}P}$ = 6.8 Hz, O**C**H$_2$CH$_3$), 62.6 (d, $^2J_{C\text{-}P}$ = 6.8 Hz, O**C**H$_2$CH$_3$), 63.0 (d, $^2J_{C\text{-}P}$ = 6.8 Hz, O**C**H$_2$CH$_3$), 63.0 (d, $^2J_{C\text{-}P}$ = 6.8 Hz, O**C**H$_2$CH$_3$), 80.8 (**C**($CH_3$)$_3$), 155.0 (**C** = O-8), 168.3 (**C** = O-4); $^{31}$P-$^1$H$_{decoup}$ NMR (121 MHz, $CDCl_3$) $\delta_P$ 24.7, 24.8; HRMS (NSI) calcd for ($C_{14}H_{28}ClN_2O_6P$), MH$^+$: 409.1266 ($^{35}$Cl), 411.1237 ($^{37}$Cl), found 409.1258 ($^{35}$Cl), 411.1231 ($^{37}$Cl). LCMS purity >95% (C-18 reversed phase, MeOH-$H_2O$).

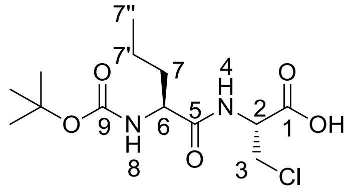

**(*R*)-2-((*S*)-2-((*tert*-Butoxycarbonyl)amino)pentanamido)-3-chloropropanoic acid or Boc-L-Nva-β-chloro-L-Ala-OH (23a)**. Deprotection of benzyl ester was followed, using (*R*)-benzyl 2-((*S*)-2-((*tert*-butoxycarbonyl)amino)pentanamido)-3-chloropropanoate (**22a**) (5.8 mmol, 2.41 g) to afford **23a** as a light yellow solid (1.81 g, 5.61 mmol, 96%); m.p. 60 - 63 °C; $\bar{\upsilon}_{max}$/cm$^{-1}$ 3312 (br OH), 2963 (NH), 1655 (br C = O), 1509 (NH bend), 1161 (C-O); $^1$H NMR (300 MHz, DMSO) $\delta_H$ 0.85 (3H, t, $^3J_{H\text{-}H}$ = 9.0 Hz, **C**H$_3$-7''), 1.24-1.34 (2H, m, **C**H$_2$-7'), 1.38 (9H. s, C(C**H**$_3$)$_3$), 1.42-1.52 (1H, m, C**H**$_{a/b}$-7), 1.54-1.59 (1H, m, C**H**$_{a/b}$-7), 3.34 (1H, br, O**H**), 3.84 (1H, dd, $^2J_{H\text{-}H}$ = 12.0 Hz, $^3J_{H\text{-}H}$ = 6.0 Hz, C**H**$_{a/b}$-3), 3.91 (1H, dd, $^2J_{H\text{-}H}$ = 12.0 Hz, $^3J_{H\text{-}H}$ = 6.0 Hz, C**H**$_{a/b}$-3), 3.95-4.02 (1H, m, C**H**-6), 4.62-4.67 (1H, m, C**H**-2), 6.92 (1H, d, $^3J_{H\text{-}H}$ = 9.0 Hz, N**H**-8), 8.07 (1H, d, $^3J_{H\text{-}H}$ = 9.0 Hz, N**H**-4); $^{13}$C NMR (75 MHz, CDCl$_3$) $\delta_C$ 14.1 (**C**H$_3$-7''), 19.1 (**C**H$_2$-7'), 28.6 (C(**C**H$_3$)$_3$), 34.4 (**C**H$_2$-7), 45.1 (**C**H$_2$-3), 53.6 (**C**H-2), 54.5 (**C**H-6), 78.5 (**C**(CH$_3$)$_3$), 155.8 (**C** = O-9), 170.6 (**C** = O-5), 173.0 (**C** = O-1); *m/z* (ESI) calcd for $(C_{13}H_{23}ClN_2NaO_5)^+$, MNa$^+$: 345.1 ($^{35}$Cl), 347.1 ($^{37}$Cl), found 345.2 ($^{35}$Cl), 347.2 ($^{37}$Cl); CHN (Found: C, 48.67; H, 7.51; N, 8.42. $C_{13}H_{23}ClN_2O_5$ requires C, 48.37; H, 7.18; N, 8.68%).

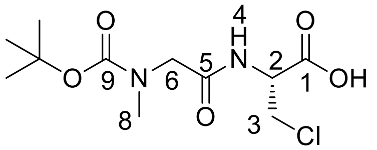

**(*R*)-2-(2-((*tert*-butoxycarbonyl)(methyl)amino)acetamido)-3-chloropropanoic acid or Boc-Sar-β-chloro-L-Ala-OH (23b)**. Deprotection of benzyl ester was followed, using (*R*)-benzyl 2-(2-((*tert*-butoxycarbonyl)(methyl)amino)acetamido)-3-chloropropanoate (**22b**) (6.2 mmol, 2.40 g) to afford **23b** as an off-white solid (1.81 g, 6.1 mmol, 99%); m.p. 89 - 91 °C; $\bar{\upsilon}_{max}$/cm$^{-1}$ 3342 (NH), 2982 (br OH), 1734 (C = O), 1672 (C = O), 1644 (C = O), 1524 (NH bend), 1152 (C-O); $^1$H NMR (300 MHz, CDCl$_3$) $\delta_H$ 1.47 (9H. s, C(C**H**$_3$)$_3$), 2.99 (3H, s, NC**H**$_3$-8), 3.81-4.17 (4H, m, C**H**$_2$-6, C**H**$_2$-3), 5.01 (1H, m, C**H**-2), 7.07 (1H, br, N**H**-4), 7.45 (1H, br, O**H**); $^{13}$C NMR (75 MHz, CDCl$_3$) $\delta_C$ 28.3 (C(**C**H$_3$)$_3$), 36.1 (N**C**H$_3$-8), 44.5 (**C**H$_2$-3), 53.0 (**C**H$_2$-6 and **C**H-2), 81.8 (**C**(CH$_3$)$_3$), 156.8 (**C** = O-9), 169.5 (**C** = O-1 and **C** = O-5); *m/z* (ESI) calcd for $(C_{11}H_{19}ClN_2NaO_5)^+$, MNa$^+$: 317.1 ($^{35}$Cl), 319.1 ($^{37}$Cl), found 317.1 ($^{35}$Cl), 319.1 ($^{37}$Cl); CHN (Found: C, 43.98; H, 6.69; N, 9.53. $C_{11}H_{19}ClN_2O_5 \cdot 0.3H_2O$ requires C, 44.02; H, 6.58; N, 9.33%).

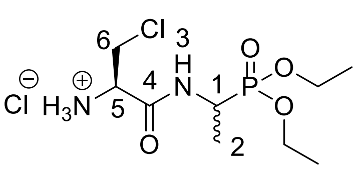

**(2*R*)-3-Chloro-1-((1-(diethoxyphosphoryl)ethyl)amino)-1-oxopropan-2-aminium chloride or β-Cl-L-Ala-D/L-Fos diethyl ester hydrochloride (20e)**. Deprotection of *tert*-butoxycarbonyl was followed, using ((2*R*)-3-chloro-1-((1-(diethoxyphosphoryl)ethyl)amino)-1-oxopropan-2-yl)carbamate (**19e**) (6.7 mmol, 2.59 g). The off-white hygroscopic crude solid was washed with petrol to afford **20e** as a pale green solid composed of 2 diastereoisomers, β-Cl-L-Ala-L-Fos diethyl ester hydrochloride and β-Cl-L-Ala-D-Fos diethyl ester hydrochloride (1.51 g, 4.7 mmol, 70%); m.p. 129 – 133 °C (decomp.); $\bar{\upsilon}_{max}$/cm$^{-1}$ 3204 (NH$^+$), 1687 (C = O), 1562 (NH bend), 1204 (P = O), 1010 (P-O-C), 961 (P-O-C); $^1$H NMR (300 MHz, D$_2$O) $\delta_H$ 1.28 (3H, t, $^3J_{H\text{-}H}$ = 6.0 Hz, OCH$_2$C**H**$_3$), 1.29 (3H, t, $^3J_{H\text{-}H}$ = 6.0 Hz, OCH$_2$C**H**$_3$), 1.37 (3H, dd, $^3J_{P\text{-}H}$ = 18.0 Hz, $^3J_{H\text{-}H}$ = 6.0 Hz, C**H**$_3$-2), 3.92-4.04 (2H, m, C**H**$_2$-6), 4.07-4.21 (4H, m, 2 x OC**H**$_2$CH$_3$), 4.38-4.48 (2H, m, C**H**-1, C**H**-5); $^{13}$C NMR (75 MHz, D$_2$O) $\delta_C$ 13.7 (**C**H$_3$-2), 14.0 (**C**H$_3$-2), 15.7 (OCH$_2$**C**H$_3$), 15.7 (OCH$_2$**C**H$_3$), 41.7 (d, $^1J_{P\text{-}C}$ = 158.3 Hz, **C**H-1), 42.0 (d, $^1J_{P\text{-}C}$ = 157.5 Hz, **C**H-1), 42.4 (**C**H$_2$-6), 53.7 (**C**H-5), 53.8 (**C**H-5), 64.3 (d, $^2J_{P\text{-}C}$ = 6.8 Hz, O**C**H$_2$CH$_3$), 64.5 (d, $^2J_{P\text{-}C}$ = 6.8 Hz, O**C**H$_2$CH$_3$), 165.7 (**C** = O-4), 165.8 (**C** = O-4); $^{31}$P-$^1$H$_{decoup}$ NMR (121 MHz, CDCl$_3$) $\delta_P$ 26.1, 26.2; HRMS (NSI) calcd for

($C_9H_{21}ClN_2O_4P$)$^+$, M$^+$: 287.0922 ($^{35}$Cl), 289.0892 ($^{37}$Cl), found 287.0922 ($^{35}$Cl), 289.0890 ($^{37}$Cl). LCMS purity >95% (C-18 reversed phase, MeOH-$H_2O$).

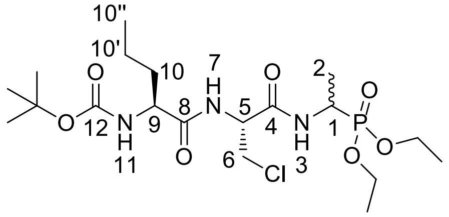

***Tert*-butyl ((2*S*)-1-(((2*R*)-3-chloro-1-((1-(diethoxyphosphoryl)-ethyl)amino)-1-oxopropan-2-yl)amino)-1-oxopentan-2-yl)carbamate or Boc-L-Nva-β-chloro-L-Ala-D/L-Fos diethyl ester (24a)**. General peptide coupling method was followed, using (*R*)-2-((*S*)-2-((*tert*-butoxycarbonyl)amino)pentanamido)-3-chloropropanoic acid (**23a**) (1.8 mmol, 0.58 g) in dry THF and diethyl 1-aminoethylphosphonate (**9**) (1.8 mmol, 0.33 g) in dry THF. The light yellow crude liquid was purified by column chromatography, using ethyl acetate/methanol (96:4), to afford **24a** as a white solid composed of 2 diastereoisomers, Boc-L-Nva-β-Cl-L-Ala-L-Fos diethyl ester and Boc-L-Nva-β-Cl-L-Ala-D-Fos diethyl ester (0.45 g, 0.93 mmol, 52%); m.p. 196 °C (decomp); $\bar{\upsilon}_{max}$/cm$^{-1}$ 3272 (NH), 1709 (C = O), 1680 (C = O), 1644 (C = O), 1530 (NH bend), 1229 (P = O), 1165 (C-O), 1019 (P-O-C), 972 (P-O-C); $^1$H NMR (300 MHz, $CDCl_3$) $\delta_H$ 0.86 (1.5H, t, $^3J_{H\text{-}H}$ = 9.0 Hz, C**H**$_3$-10′′), 0.88 (1.5H, t, $^3J_{H\text{-}H}$ = 9.0 Hz, C**H**$_3$-10′′), 1.22-1.34 (11H, m, 2 x $OCH_2$C**H**$_3$, C**H**$_3$-2, C**H**$_2$-10′), 1.38 (9H, s, C(C**H**$_3$)$_3$), 1.53-1.59 (1H, m, C**H**$_{a/b}$-10), 1.70-1.77 (1H, m, C**H**$_{a/b}$-10), 3.69 (1H, dd, $^2J_{H\text{-}H}$ = 12.0 Hz, $^3J_{H\text{-}H}$ = 6.0 Hz, C**H**$_{a/b}$-6), 3.78-3.81 (1H, m, C**H**-9), 3.91 (1H, dd, $^2J_{H\text{-}H}$ = 12.0 Hz, $^3J_{H\text{-}H}$ = 6.0 Hz, C**H**$_{a/b}$-6), 3.97-4.13 (4H, m, 2 x OC**H**$_2$CH$_3$), 4.35-4.46 (1H, m, C**H**-1), 4.73-4.79 (1H, m, C**H**-5), 4.97-5.03 (1H, m, N**H**-11), 7.01 (0.5H, d, $^3J_{H\text{-}H}$ = 9.0 Hz, N**H**-7), 7.09 (0.5H, d, $^3J_{H\text{-}H}$ = 9.0 Hz, N**H**-7), 7.25 (0.5H, d, $^3J_{H\text{-}H}$ = 9.0 Hz, N**H**-3), 7.33 (0.5H, d, $^3J_{H\text{-}H}$ = 9.0 Hz, N**H**-3); $^{13}$C NMR (75 MHz, $CDCl_3$) $\delta_C$ 12.7 (**C**H$_3$-10′′), 14.5 (**C**H$_3$-2), 15.3 ($OCH_2$**C**H$_3$), 15.4 ($OCH_2$**C**H$_3$), 15.5 ($OCH_2$**C**H$_3$), 15.6 ($OCH_2$**C**H$_3$), 17.9 (**C**H$_2$-10′), 18.0 (**C**H$_2$-10′), 27.0 (C(**C**H$_3$)$_3$), 27.3 (C(**C**H$_3$)$_3$), 33.2 (**C**H$_2$-10), 40.4 (d, $^1J_{C\text{-}P}$ = 157.5 Hz, **C**H-1), 43.4 (**C**H$_2$-6), 52.6 (**C**H-5), 52.8 (**C**H-5), 61.4 (d, $^2J_{C\text{-}P}$ = 6.8 Hz, O**C**H$_2$CH$_3$), 61.6 (d, $^2J_{C\text{-}P}$ = 6.8 Hz, O**C**H$_2$CH$_3$), 61.7 (d, $^2J_{C\text{-}P}$ = 6.8 Hz, O**C**H$_2$CH$_3$), 61.9 (d, $^2J_{C\text{-}P}$ = 6.8 Hz, O**C**H$_2$CH$_3$), 70.5 (**C**H-9), 79.4 (**C**(CH$_3$)$_3$), 154.7 (**C** = O-12), 166.8 (**C** = O-4), 171.4 (**C** = O-8); $^{31}$P-$^1$H$_{decoup}$ NMR (121 MHz, $CDCl_3$) $\delta_P$ 24.9, 25.0; HRMS (NSI) calcd for ($C_{19}H_{38}ClN_3O_7P$)$^+$, MH$^+$: 486.2130 ($^{35}$Cl), 488.2102 ($^{37}$Cl), found 486.2124 ($^{35}$Cl), 488.2098 ($^{37}$Cl); CHN (Found: C, 46.61; H, 7.76; N, 8.31. $C_{19}H_{37}ClN_3O_7P$ requires C, 46.96; H, 7.67; N, 8.65%).

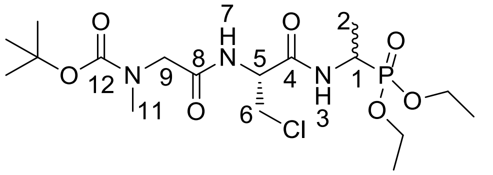

***Tert*-butyl (2-(((2*R*)-3-chloro-1-((1-(diethoxyphosphoryl)ethyl) amino)-1-oxopropan-2-yl)amino)-2-oxoethyl)(methyl)carbamate or Boc-Sar-β-chloro-L-Ala-D/L-Fos diethyl ester (24b)**. General peptide coupling method was followed, using (*R*)-2-(2-((*tert*-butoxycarbonyl)(methyl)amino)acetamido)-3-chloropropanoic acid (**23b**) (5.5 mmol, 1.61 g) in dry THF and diethyl 1-aminoethylphosphonate (**9**) (6.0 mmol, 1.09 g) in dry THF. The light yellow crude liquid was purified by column chromatography, using DCM/methanol (95:5), to afford **24b** as a light yellow syrup composed of 2 diastereoisomers, Boc-Sar-β-Cl-L-Ala-L-Fos diethyl ester and Boc-Sar-β-Cl-L-Ala-D-Fos diethyl ester (1.93 g, 4.21 mmol, 76%); $\bar{\upsilon}_{max}$/cm$^{-1}$ 3218 (NH), 1690 (C = O), 1665 (br C = O), 1518 (NH bend), 1224 (P = O), 1148 (C-O), 1018 (P-O-C), 967 (P-O-C); $^1$H NMR (300 MHz, $CDCl_3$) $\delta_H$ 1.11-1.35 (9H, m, 2 x $OCH_2$C**H**$_3$, C**H**$_3$-2), 1.41 (9H, s, C(C**H**$_3$)$_3$), 2.90 (3H, s, C**H**$_3$-11), 3.70-3.88 (4H, m, C**H**$_2$-6, C**H**$_2$-9), 4.02-4.13 (4H, m, 2 x OC**H**$_2$CH$_3$), 4.36-4.47 (1H, m, C**H**-1), 4.78-4.82 (1H, m, C**H**-5), 6.94 (1H, m, N**H**-7), 7.36 (1H, m, N**H**-3); $^{13}$C NMR (75 MHz, $CDCl_3$) $\delta_C$ 15.2 (**C**H$_3$-2), 15.6 (**C**H$_3$-2), 16.3 ($OCH_2$**C**H$_3$), 16.4 ($OCH_2$**C**H$_3$), 28.3 (C(**C**H$_3$)$_3$), 35.9 (**C**H$_3$-11), 41.2 (d, $^1J_{C\text{-}P}$ = 156.8 Hz, **C**H-1), 44.7 (**C**H$_2$-6), 53.1 (**C**H$_2$-9), 53.4 (**C**H-5), 62.7 (d, $^2J_{C\text{-}P}$ = 6.0 Hz, O**C**H$_2$CH$_3$), 63.0 (d, $^2J_{C\text{-}P}$ = 7.5 Hz, O**C**H$_2$CH$_3$), 81.0 (**C**(CH$_3$)$_3$), 152.3 (**C** = O-12), 167.7 (**C** = O-4 or **C** = O-8), 169.4 (**C** = O-4 or **C** = O-8); $^{31}$P-$^1$H$_{decoup}$ NMR

(121 MHz, $CDCl_3$) $\delta_P$ 24.7, 24.8; HRMS (NSI) calcd for $(C_{16}H_{34}ClN_3O_7P)^+$, $MH^+$: 480.1637 ($^{35}Cl$), 482.1608 ($^{37}Cl$), found 480.1642 ($^{35}Cl$), 482.1612 ($^{37}Cl$). LCMS purity >92% (C-18 reversed phase, MeOH-$H_2O$)

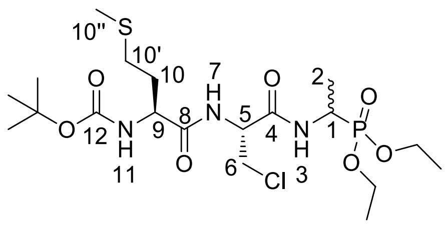

***Tert*-butyl ((2*S*)-1-(((2*R*)-3-chloro-1-((1-(diethoxyphosphoryl) ethyl)amino)-1-oxopropan-2-yl)amino)-4-(methylthio)-1-oxobutan-2-yl) carbamate or Boc-L-Met-β-Cl-L-Ala-D/L-Fos diethyl ester (24c)**. General peptide coupling method was followed, using Boc-L-Met-OH (**15c**) (3.4 mmol, 0.85 g) in dry THF and (2*R*)-3-chloro-1-((1-(diethoxyphosphoryl)ethyl)amino)-1-oxopropan-2-aminium chloride (**20e**) (3.4 mmol, 1.10 g) in dry DCM. The yellow crude liquid was purified by column chromatography (DCM/MeOH (95:5)) and recrystallized from diethyl ether/ petrol to give **24c** as a white solid composed of 2 diastereoisomers, Boc-L-Met-β-Cl-L-Ala-L-Fos diethyl ester and Boc-L-Met-β-Cl-L-Ala-D-Fos diethyl ester (0.88 g, 1.7 mmol, 50%); m.p. 96 – 99 °C; $\bar{\upsilon}_{max}$/cm$^{-1}$ 3278 (NH), 1709 (C = O), 1687 (C = O), 1639 (C = O), 1523 (NH bend), 1228 (P = O), 1165 (C-O), 1018 (P-O-C), 970 (P-O-C); $^1H$ NMR (300 MHz, $CDCl_3$) $\delta_H$ 1.17-1.36 (9H, m, C**H**$_3$-2, 2 x $OCH_2CH_3$), 1.38 (9H, s, C(C**H**$_3$)$_3$), 1.87-2.03 (2H, m, C**H**$_2$-10), 2.04 (3H, s, C**H**$_3$-10′′), 2.48-2.54 (2H, m, C**H**$_2$-10′), 3.71 (1H, dd, $^2J_{H-H}$ = 12.0 Hz, $^3J_{H-H}$ = 6.0 Hz, C**H**$_{a/b}$-6), 3.88 (1H, dd, $^2J_{H-H}$ = 12.0 Hz, $^3J_{H-H}$ = 6.0 Hz, C**H**$_{a/b}$-6), 3.99-4.13 (4H, m, 2 x OC**H**$_2$CH$_3$), 4.20 (1H, m, C**H**-9), 4.37-4.47 (1H, m, C**H**-1), 4.78-4.84 (1H, m, C**H**-5), 5.39 (0.5H, d, $^3J_{H-H}$ = 6.0 Hz, N**H**-11), 5.41 (0.5H, d, $^3J_{H-H}$ = 6.0 Hz, N**H**-11), 7.15 (0.5H, d, $^3J_{H-H}$ = 6.0 Hz, N**H**-7), 7.24 (0.5H, d, $^3J_{H-H}$ = 6.0 Hz, N**H**-7), 7.52 (1H, m, N**H**-3); $^{13}C$ NMR (75 MHz, $CDCl_3$) $\delta_C$ 14.3 (**C**H$_3$-2), 14.4 (**C**H$_3$-2), 14.5 (**C**H$_2$-10′′), 15.3 (OCH$_2$**C**H$_3$), 15.4 (OCH$_2$**C**H$_3$), 15.5 (OCH$_2$**C**H$_3$), 15.6 (OCH$_2$**C**H$_3$), 27.3 (C(**C**H$_3$)$_3$), 29.2 (**C**H$_2$-10′), 29.3 (**C**H$_2$-10′), 30.2 (**C**H$_2$-10), 30.4 (**C**H$_2$-10), 40.3 (d, $^1J_{P-C}$ = 159.0 Hz, **C**H-1), 43.5 (**C**H$_2$-6), 43.7 (**C**H$_2$-6), 52.7 (**C**H-5), 53.1 (**C**H-9), 61.6 (d, $^2J_{P-C}$ = 6.8 Hz, O**C**H$_2$CH$_3$), 61.7 (d, $^2J_{P-C}$ = 6.0 Hz, O**C**H$_2$CH$_3$), 62.0 (d, $^2J_{P-C}$ = 6.8 Hz, O**C**H$_2$CH$_3$), 62.1 (d, $^2J_{P-C}$ = 7.5 Hz, O**C**H$_2$CH$_3$), 79.6 (**C**(CH$_3$)$_3$), 154.8 (**C** = O-12), 166.7 (**C** = O-4), 166.8 (**C** = O-4), 170.7 (**C** = O-8), 170.8 (**C** = O-8); $^{31}P$-$^1H_{decoup}$ NMR (121 MHz, $CDCl_3$) $\delta_P$ 24.5, 24.8; HRMS (NSI) calcd for $(C_{19}H_{3H}ClN_3O_7PS)^{+,}$ $MH^+$: 518.1851 ($^{35}Cl$), 520.1821 ($^{37}Cl$), found 518.1842 ($^{35}Cl$), 520.1814 ($^{37}Cl$); CHN (Found: C, 44.08; H, 7.47; N, 8.18. $C_{19}H_{37}ClN_3O_7PS$ requires C, 44.06; H, 7.20; N, 8.11%).

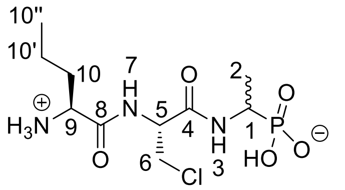

**(1-((*R*)-2-((*S*)-2-Ammoniopentanamido)-3-chloropropanamido) ethyl)phosphonic acid or L-Nva-β-chloro-L-Ala-D/L-Fos (25a)**. The *tert*-butoxycarbonyl and diethyl ester protecting groups of *tert*-butyl ((2*S*)-1-(((2*R*)-3-chloro-1-((1-(diethoxyphosphoryl)-ethyl)amino)-1-oxopropan-2-yl)amino)-1-oxopentan-2-yl)carbamate (**24a**) (2.0 mmol, 0.99 g) were removed. The pale green crude solid was washed with diethyl ether to give **25a** as a pale green solid composed of 2 diastereoisomers, L-Nva-β-Cl-L-Ala-L-Fos and L-Nva-β-Cl-L-Ala-D-Fos (0.64 g, 1.94 mmol, 97%); m.p. 175 °C (sub); $\bar{\upsilon}_{max}$/cm$^{-1}$ 3294 ($NH^+$), 3000 (br OH), 1668 (C = O), 1645 (C = O), 1538 (NH bend), 1132 (P = O), 1039 (P-O-C), 921 (P-OH); $^1H$ NMR (300 MHz, $D_2O$) $\delta_H$ 1.01 (3H, t, $^3J_{H-H}$ = 9.0 Hz, C**H**$_3$-10′′), 1.30-1.37 (3H, br m, C**H**$_3$-2), 1.44-1.54 (2H, br m C**H**$_2$-10′), 1.90-1.98 (2H, br m, C**H**$_2$-10), 3.91-4.15 (4H, br m, C**H**$_2$-6, C**H**-9, C**H**-1), 4.79 (1H, br m, C**H**-5); $^{13}C$ NMR (75 MHz, $D_2O$) $\delta_C$ 12.9 (**C**H$_3$-10′′), 15.7 (**C**H$_3$-2), 17.6 (**C**H$_2$-10′), 33.0 (**C**H$_2$-10), 43.3 (**C**H$_2$-6), 53.1 (**C**H-1 and **C**H-9), 55.0 (**C**H-5), 170.4 (**C** = O-4 and **C** = O-8); $^{31}P$-$^1H_{decoup}$ NMR (121 MHz, $CDCl_3$) $\delta_P$ 18.5; HRMS (NSI) calcd for $(C_{10}H_{20}ClN_3O_5P)^{-,}$ $MH^-$: 328.0835 ($^{35}Cl$), 330.0805 ($^{37}Cl$), found 328.0833 ($^{35}Cl$), 330.0800 ($^{37}Cl$). LCMS purity >95% (C-18 reversed phase, MeOH-$H_2O$).

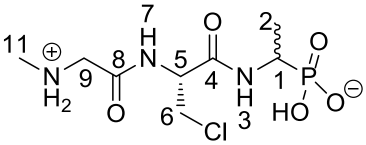

**1-((*R*)-3-Chloro-2-(2-(methylammonio)acetamido) propanamido)ethyl)phosphonic acid or Sar-β-chloro-L-Ala-D/L-Fos (25b)**. The *tert*-butoxycarbonyl and diethyl ester protecting groups of *tert*-butyl (2-(((2*R*)-3-chloro-1-((1-(diethoxyphosphoryl)ethyl)amino)-1-oxopropan-2-yl)amino)-2-oxoethyl)(methyl)carbamate (**24b**) (3.8 mmol, 1.74 g) were removed. The pale green crude solid was recrystallised from hot water/ethanol to give **25b** as an off-white solid composed of 2 diastereoisomers, Sar-β-Cl-L-Ala-L-Fos and Sar-β-Cl-L-Ala-D-Fos (0.49 g, 1.61 mmol, 42%); m.p. 185-188 °C (decomp.); $\bar{\upsilon}_{max}$/cm$^{-1}$ 3287 ($NH^+$), 3000 (br OH), 1657 (C = O), 1634 (C = O), 1552 (NH bend), 1172 (P = O), 1054 (P-O-C), 919 (P-OH); $^1$H NMR (300 MHz, $CD_3OD$) $\delta_H$ 1.24 (3H, dd, $^3J_{H\text{-}P}$ = 15.0 Hz, $^3J_{H\text{-}H}$ = 6.0 Hz, C**H**$_3$-2), 2.74 (3H, s, NC**H**$_3$-11), 3.81-3.87 (2H, m, C**H**$_2$-6), 3.93-3.94 (2H, m, C**H**$_2$-9), 3.97-4.10 (1H, m, C**H**-1), 4.79 (1H, m, C**H**-5); $^{13}$C NMR (75 MHz, $CD_3OD$) $\delta_C$ 15.4 (**C**H$_3$-2), 32.9 (N**C**H$_3$-11), 43.6 (**C**H$_2$-6), 44.1 (d, $^1J_{C\text{-}P}$ = 148.5 Hz, **C**H-1), 49.5 (**C**H$_2$-9), 54.6 (**C**H-5), 166.4 (**C** = O-4 or **C** = O-9), 166.9 (**C** = O-4 or **C** = O-9); $^{31}$P-$^1$H$_{decoup}$ NMR (121 MHz, $CDCl_3$) $\delta_P$ 18.8; HRMS (NSI) calcd for ($C_8H_{18}ClN_3O_5P$)$^{+,}$ MH$^+$: 302.0667 ($^{35}$Cl), 304.0638 ($^{37}$Cl), found 302.0670 ($^{35}$Cl), 304.0640 ($^{37}$Cl). LCMS purity >95% (C-18 reversed phase, MeOH-$H_2O$).

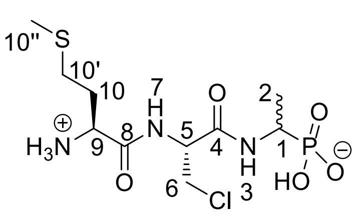

**(1-((*R*)-2-((*S*)-2-Ammonio-4-(methylthio)butanamido)-3-chloro propanamido)ethyl)phosphonic acid or L-Met-β-Cl-L-Ala-D/L-Fos (25c)**. The *tert*-butoxycarbonyl and diethyl ester protecting groups of *tert*-butyl ((2*S*)-1-(((2*R*)-3-chloro-1-((1-(diethoxyphosphoryl) ethyl)amino)-1-oxopropan-2-yl)amino)-4-(methylthio)-1-oxobutan-2-yl)carbamate (**24c**) (1.4 mmol, 0.71 g) were removed. The green crude solid was recrystallised from hot water/ethanol to give **25c** as a pale green solid composed of 2 diastereoisomers, L-Met-β-Cl-L-Ala-L-Fos and L-Met-β-Cl-L-Ala-D-Fos (0.17 g, 0.48 mmol, 35%); m.p. 175 – 179 °C (decomp.); $\bar{\upsilon}_{max}$/cm$^{-1}$ 3264 ($NH^+$), 2829 (broad OH), 1666(C = O), 1641 (C = O), 1546 (NH bend), 1149 (P = O), 1041 (P-O-C), 921 (P-OH); $^1$H NMR (300 MHz, $D_2O$) $\delta_H$ 1.31 (3H, dd, $^3J_{H\text{-}P}$ = 15.0 Hz, $^3J_{H\text{-}H}$ = 6.0 Hz, C**H**$_3$-2), 2.13 (3H, s, C**H**$_3$-10′′), 2.18-2.29 (2H, m, C**H**$_2$-10), 2.63-2.69 (2H, m, C**H**$_2$-10′), 3.89 (1H, dd, $^2J_{H\text{-}H}$ = 12.0 Hz, $^3J_{H\text{-}H}$ = 6.0 Hz, C**H**$_{a/b}$-6), 3.97 (1H, dd, $^2J_{H\text{-}H}$ = 12.0 Hz, $^3J_{H\text{-}H}$ = 6.0 Hz, C**H**$_{a/b}$-6), 4.01-4.13 (1H, m, C**H**-1), 4.22 (1H, br m, C**H**-9), 4.75-4.79 (1H, m, C**H**-5); $^{13}$C NMR (75 MHz, $D_2O$) $\delta_C$ 16.9 (**C**H$_3$-10′′), 17.0 (**C**H$_3$-10′′), 18.4 (**C**H$_3$-2), 31.1 (**C**H$_2$-10′), 32.9 (**C**H$_2$-10), 46.2 (**C**H$_2$-6), 47.0 (d, $^1J_{C\text{-}P}$ = 147.0 Hz, **C**H-1), 52.2 (**C**H-9), 52.3 (**C**H-9), 57.8 (**C**H-5), 58.0 (**C**H-5), 171.7 (**C** = O-4), 171.8 (**C** = O-4), 172.3 (**C** = O-8); $^{31}$P-$^1$H$_{decoup}$ NMR (121 MHz, $CDCl_3$) $\delta_P$ 18.7; HRMS (NSI) calcd for ($C_{10}H_{21}ClN_3O_5PS$), MNa$^+$: 384.0520 ($^{35}$Cl), 386.0489 ($^{37}$Cl), found 384.0523 ($^{35}$Cl), 386.0491 ($^{37}$Cl). LCMS purity >95% (C-18 reversed phase, MeOH-$H_2O$).

**Table S1:** Summary of yield and ratio of diastereoisomers and enantiomers

| Compounds | | Yield (%) | Ratio of Diastereoisomers/Enantiomers |
|---|---|---|---|
| **D/L-fosfalin** | **2-DL** | 98 | 1: 1 based on optical rotation |
| **Trifluoroacetyl-D/L-Fos diethyl ester** | **8** | 80 | 1: 1 based on optical rotation |
| **D/L-Fos diethyl ester** | **9** | 97 | 1: 1 based on optical rotation |
| **Boc-L-Ser-OBzl** | **11** | 89 | NM |
| **Boc-β-Cl-L-Ala-OBzl** | **12** | 75 | NM |
| **Boc-β-Cl-L-Ala-OH** | **13** | 97 | NM |

| | | | |
|---|---|---|---|
| **β-Cl-L-Ala-OBzl hydrochloride** | **14** | 93 | NM |
| **Boc-L-Nva-L-Ala-OBzl** | **16a** | 63 | NM |
| **Boc-Sar-L-Ala-OBzl** | **16b** | 75 | NM |
| **Boc-L-Nva-L-Ala-OH** | **17a** | 96 | NM |
| **Boc-Sar-L-Ala-OH** | **17b** | 96 | NM |
| **Boc-L-Nva-L-Ala-D/L-Fos diethyl ester** | **18a** | 78 | ND |
| **Boc-Sar-L-Ala-D/L-Fos** | **18b** | 63 | ND |
| **Boc-L-Met-L-Ala-D/L-Fos diethyl ester** | **18c** | 32 | ND |
| **Boc-L-Ala-D/L-Fos diethyl ester** | **19d** | 71 | ND |
| **Boc-β-chloro-L-Ala-D/L-Fos diethyl ester** | **19e** | 88 | ND |
| **L-Ala-D/L-Fos diethyl ester hydrochloride** | **20d** | 84 | 1: 1.2 based on LC-MS peak area |
| **β-Cl-L-Ala-D/L-Fos diethyl ester hydrochloride** | **20e** | 70 | 1: 1.4 based LC-MS peak area |
| **L-Nva-L-Ala-D/L-Fos** | **21a** | 47 | 1: 1.3 based on LC-MS peak area |
| **Sar-L-Ala-D/L-Fos** | **21b** | 51 | 1: 1.4 based on LC-MS peak area |
| **L-Met-L-Ala-D/L-Fos** | **21c** | 46 | 1: 1.8 based on LC-MS peak area |
| **Boc-L-Nva-β-chloro-L-Ala-OBzl** | **22a** | 78 | NM |
| **Boc-Sar-β-chloro-L-Ala-OBzl** | **22b** | 73 | NM |
| **Boc-L-Nva-β-chloro-L-Ala-OH** | **23a** | 96 | NM |
| **Boc-Sar-β-chloro-L-Ala-OH** | **23b** | 99 | NM |
| **Boc-L-Nva-β-chloro-L-Ala-D/L-Fos diethyl ester** | **24a** | 52 | ND |
| **Boc-Sar-β-chloro-L-Ala-D/L-Fos diethyl ester** | **24b** | 76 | ND |
| **Boc-L-Met-β-Cl-L-Ala-D/L-Fos diethyl ester** | **24c** | 50 | ND |
| **L-Nva-β-chloro-L-Ala-D/L-Fos** | **25a** | 97 | 1: 1.8 based on LC-MS peak area |
| **Sar-β-chloro-L-Ala-D/L-Fos** | **25b** | 42 | 1: 1.6 based on LC-MS peak area |
| **L-Met-β-Cl-L-Ala-D/L-Fos** | **25c** | 35 | 1: 1 based on LC-MS peak area |

Note: Ratio of diastereoisomers/enantiomers was obtained from optical rotation or LC-MS experiment. Assignment of ratio to the corresponding diastereoisomers, L,L and L,D, L,L,L and L,L,D was impossible without standards. Optical rotation at 0° showed compounds are racemic with 1:1 ratio. NM: Not measured because NMR spectra did not show diastereoisomeric coupling. ND: Not determined because diastereoisomeric peaks were only detected by LC-MS upon removal of protecting group from either the *N*- and/or *O*-terminus of phosphonotripeptide derivatives.

## 2. NMR spectra

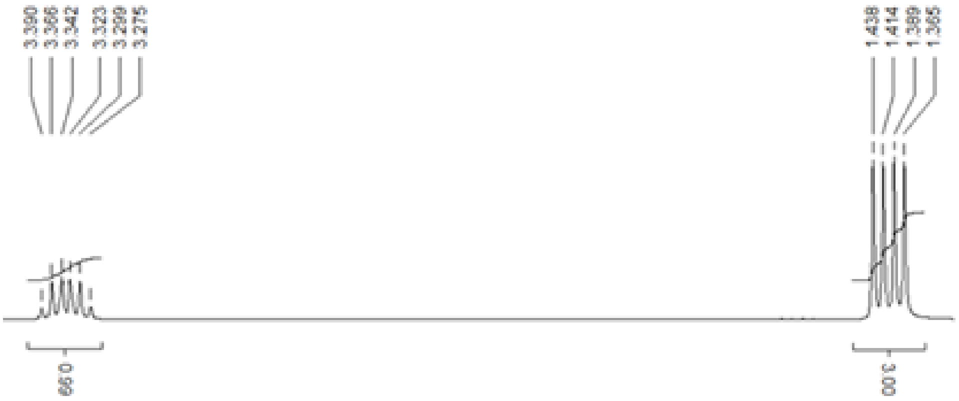


**Figure S1**. $^{1}$H-NMR spectrum of fosfalin **2-DL** in $D_2O$ at 300 MHz.

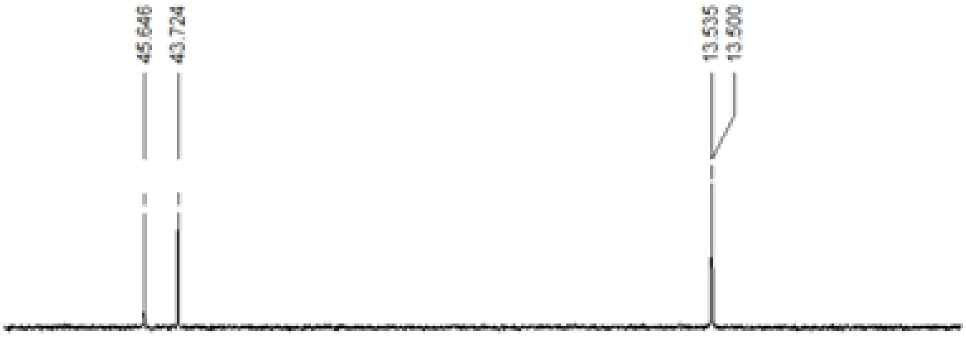


**Figure S2**. $^{13}$C-NMR spectrum of fosfalin **2-DL** in $D_2O$ at 300 MHz.

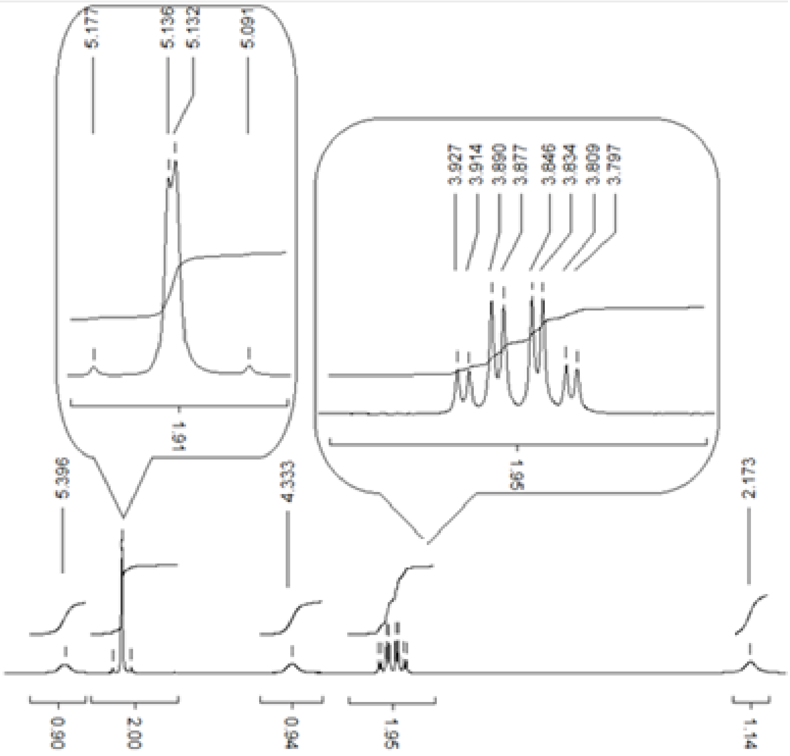


**Figure S3**. $^{1}$H-NMR spectrum of Boc-L-Ser-OBzl **11** in $CDCl_3$ at 300 MHz, range $\delta_H$ 2.10 - 5.40 ppm.

## 3. Low Resolution MS spectrum

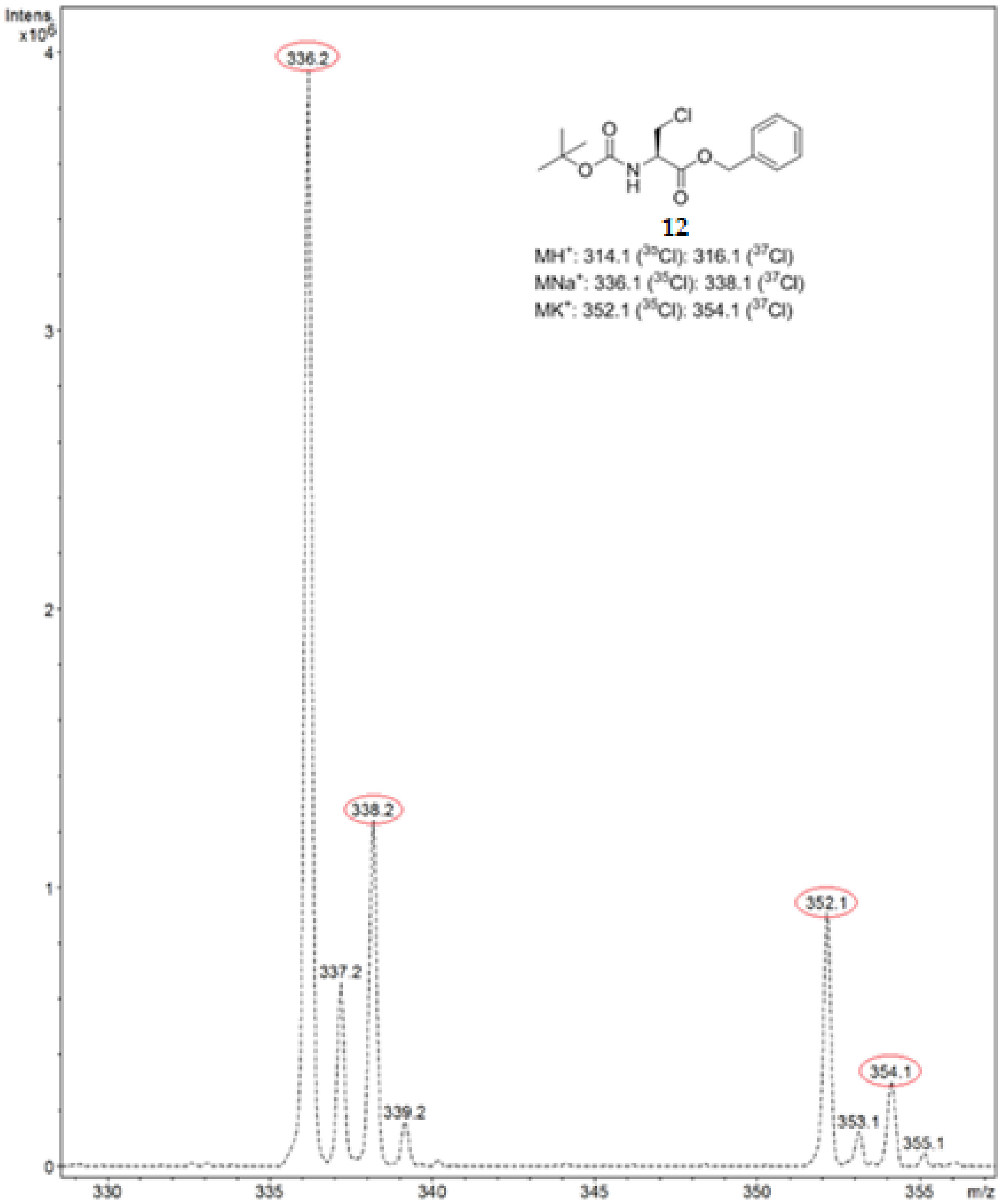


**Figure S4**. Low resolution MS spectrum of Boc-β-chloro-L-Ala-OBzl **12**, showing the 3:1 ratio of $^{35}$Cl:$^{37}$Cl.

## 4. LC-MS Conditions

LC-MS analysis was performed using an Agilent 1290 Infinity Series HPLC system and an Agilent 6120 Quadrupole LC-MS detector. ACE Excel 5 Super C18 (150 x 4.6 mm i.d.) LC column was used. LC-MS data was analysed by Agilent ChemStation.
Mobile phase: water and methanol (95:5) + 0.1% formic acid
Flow time: 0.75 mL/min
Injection volume: 10 μL
Column temperature: 35 °C
Vial temperature: 25 °C
Sample concentration: 0.1 mg/mL in mobile phase

### *4.1 LC-MS Chromatograms*

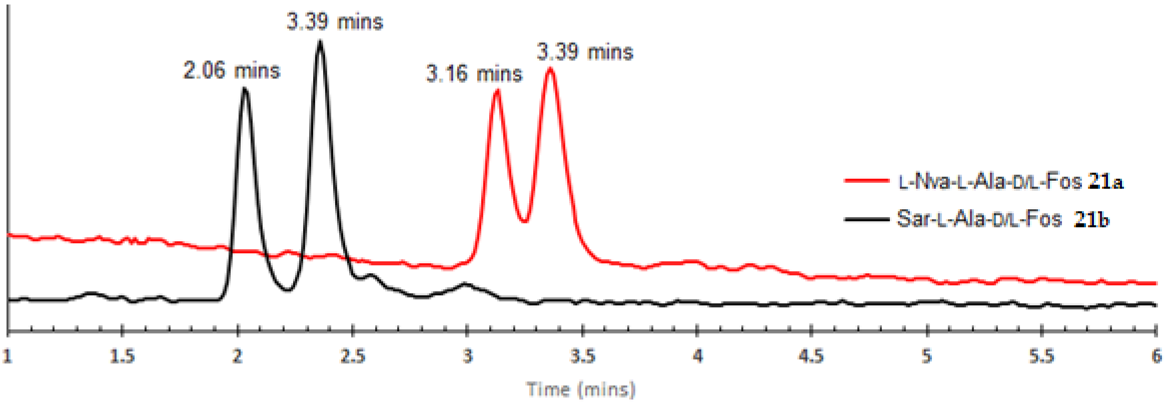


**Figure S5**. Reversed phase LC-MS chromatograms of L-Nva-L-Ala-D/L-Fos **21a** and Sar-L-Ala-D/L-Fos **21b** with specific ions extracted at MH+ *m/z* 296 and *m/z* 268, respectively. For clarity, the chromatograms are displayed between the range 1.0 - 6.0 mins.

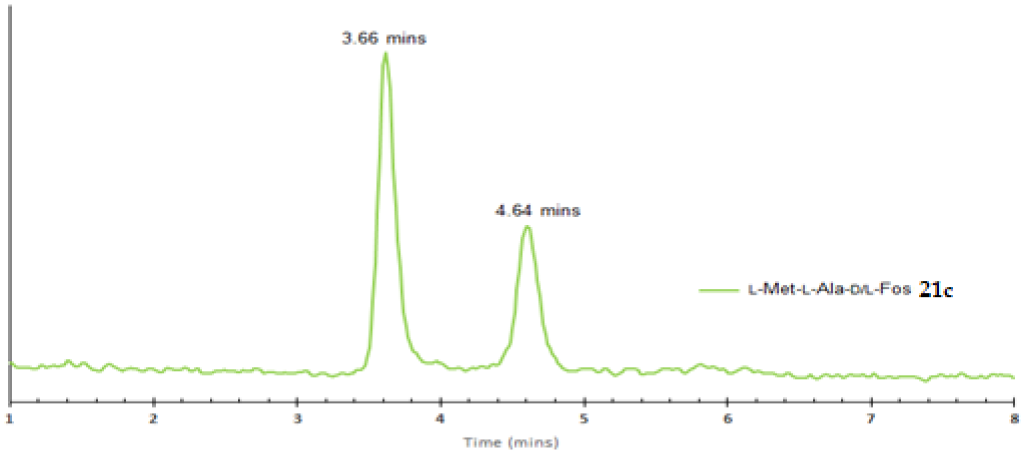


**Figure S6**. Reversed phase LC-MS chromatograms of L-Met-L-Ala-D/L-Fos **21c** with specific ions extracted at MH+ *m/z* 328. For clarity, the chromatograms are displayed between the range 1.0 - 8.0 mins.

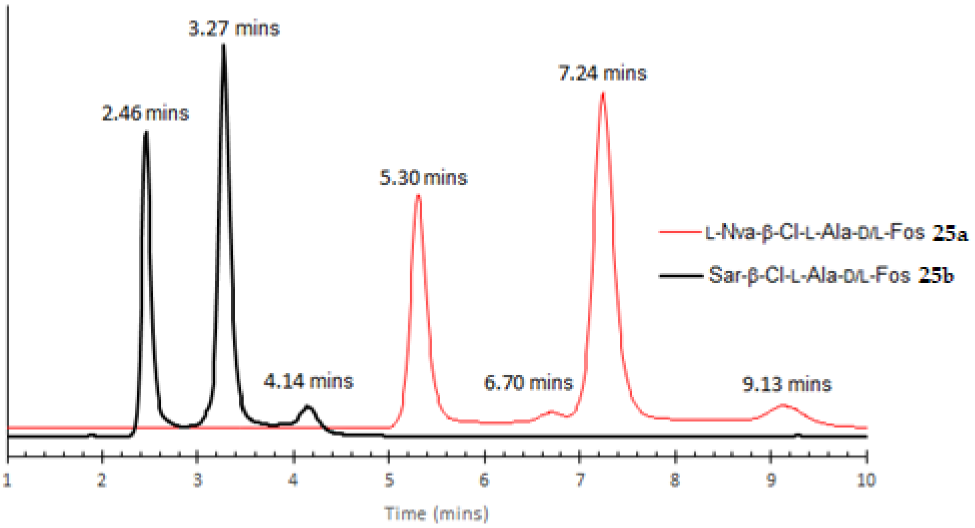


**Figure S7**. Reversed phase LC-MS chromatograms of L-Nva-β-chloro-L-Ala-D/L-Fos **25a** and Sar-β-chloro-L-Ala-D/L-Fos **25b** with specific ions extracted at MH+ *m*/*z* 330 and *m*/*z* 302, respectively. For clarity, the chromatograms are displayed between the range 1.0 - 10.0 mins.

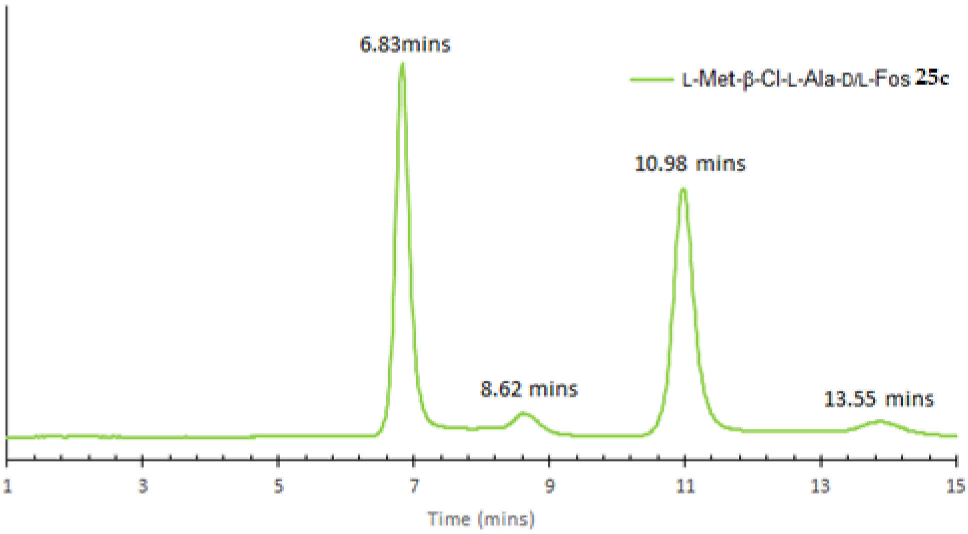


**Figure S8**. Reversed phase LC-MS chromatograms of L-Met-β-chloro-L-Ala-D/L-Fos **25c** with specific ions extracted at MH+ *m*/*z* 362. For clarity, the chromatograms are displayed between the range 1.0–15.0 mins.
